# Glycogen phosphorylase inhibition improves cognitive function of aged mice

**DOI:** 10.1111/acel.13928

**Published:** 2023-07-31

**Authors:** Dominika Drulis‐Fajdasz, Adam Krzystyniak, Alicja Puścian, Agata Pytyś, Kinga Gostomska‐Pampuch, Natalia Pudełko‐Malik, Jerzy Ł. Wiśniewski, Piotr Młynarz, Arkadiusz Miazek, Tomasz Wójtowicz, Jakub Włodarczyk, Kamila Duś‐Szachniewicz, Agnieszka Gizak, Jacek R. Wiśniewski, Dariusz Rakus

**Affiliations:** ^1^ Department of Molecular Physiology and Neurobiology University of Wroclaw Wroclaw Poland; ^2^ Laboratory of Cell Biophysics Nencki Institute of Experimental Biology, Polish Academy of Sciences Warsaw Poland; ^3^ Nencki‐EMBL Partnership for Neural Plasticity and Brain Disorders – BRAINCITY Nencki Institute of Experimental Biology, Polish Academy of Sciences Warsaw Poland; ^4^ Department of Biochemistry and Immunochemistry Wroclaw Medical University Wroclaw Poland; ^5^ Biochemical Proteomics Group, Department of Proteomics and Signal Transduction Max Planck Institute of Biochemistry Martinsried Germany; ^6^ Department of Biochemistry, Molecular Biology and Biotechnology, Faculty of Chemistry Wroclaw University of Science and Technology Wroclaw Poland; ^7^ Laboratory of Tumor Immunology Hirszfeld Institute of Immunology and Experimental Therapy, Polish Academy of Sciences Wroclaw Poland; ^8^ Department of Clinical and Experimental Pathology Institute of General and Experimental Pathology, Wroclaw Medical University Wroclaw Poland

**Keywords:** aging, behavioral tests, glycogen phosphorylase (Pyg), hippocampus, memory formation/deficits

## Abstract

Inhibition of glycogen breakdown blocks memory formation in young animals, but it stimulates the maintenance of the long‐term potentiation, a cellular mechanism of memory formation, in hippocampal slices of old animals. Here, we report that a 2‐week treatment with glycogen phosphorylase inhibitor BAY U6751 alleviated memory deficits and stimulated neuroplasticity in old mice. Using the 2‐Novel Object Recognition and Novel Object Location tests, we discovered that the prolonged intraperitoneal administration of BAY U6751 improved memory formation in old mice. This was accompanied by changes in morphology of dendritic spines in hippocampal neurons, and by “rejuvenation” of hippocampal proteome. In contrast, in young animals, inhibition of glycogen degradation impaired memory formation; however, as in old mice, it did not alter significantly the morphology and density of cortical dendritic spines. Our findings provide evidence that prolonged inhibition of glycogen phosphorolysis improves memory formation of old animals. This could lead to the development of new strategies for treatment of age‐related memory deficits.

## Graphical abstract

In this paper, we present functional characterization of mice treated with the glycogen phosphorylase inhibitor BAY U6751 (BAY). We show that the BAY‐induced, prolonged inhibition of glycogen breakdown alleviates memory deficits in old mice and restores neuronal plasticity in their hippocampi. Electrophysiological tests reveal that training after the BAY‐treatment occludes LTP induction. The inhibition of glycogen breakdown is also correlated with the “rejuvenation” of hippocampal proteome.

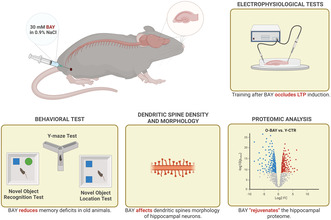

**Abbreviations**
ANLS: astrocyte‐neuronal lactate shuttle
BAY ‐ BAY U6751: 4‐(2‐Chlorophenyl)‐1‐ethyl‐1,4‐dihydro‐6‐methyl‐2,3,5‐pyridinetricarboxylic acid 5‐isopropyl
DAPs: differentially abundant proteins
LOQ: limit of quantification
LTP: long term potentiation
NMDAR: N‐methyl‐D‐aspartate receptor
NOL: novel object location test
NOR: 2‐novel object recognition test
PCA: principal component analysis
Pyg: glycogen phosphorylase
SPT: sucrose preference test
TPA: total protein approach

## 1 INTRODUCTION

Glycogen phosphorylase (Pyg) catalyzes the first and rate‐limiting step in the process of glycogen degradation (glycogenolysis). Inhibition of Pyg was shown to block memory formation in young chickens (Gibbs et al., 2006) and induction of the Long Term Potentiation (LTP, a cellular/molecular mechanism of memory formation) in the hippocampi and hippocampal slices isolated from young rodents, respectively (Drulis‐Fajdasz et al., 2015; Suzuki et al., 2011). It was also shown that impairment of synaptic plasticity after Pyg inhibition was associated with decreased transport of glycogen‐derived lactate from astrocytes to neurons in a process called the astrocyte‐neuronal lactate shuttle (ANLS; Magistretti & Allaman, 2015). The impact of the astrocytic glycogen‐derived lactate on neuronal metabolism is the subject of ongoing debate (Dienel & Cruz, 2015) and the mechanism by which this pool of lactate stimulates the LTP is not fully understood. However, it is commonly accepted that disruption of the ANLS affects memory formation (Hertz & Chen, 2018).

In contrast to the young animals, inhibition of glycogen breakdown in hippocampal sections isolated from adult and aged rodents was shown to improve the LTP formation, elevating significantly its magnitude (Drulis‐Fajdasz et al., 2015). Moreover, in hippocampal slices isolated from old animals, significant alterations in morphology of dendritic spines were observed after inhibition of Pyg, indicating changes in dendritic spines maturation (Drulis‐Fajdasz et al., 2015). Mechanisms underlying this different response to Pyg inhibition remain to be discovered but they might be associated with a different organization of hippocampal formation in young and aged animals (Drulis‐Fajdasz et al., 2018) and global changes in the expression of hippocampal proteins (Drulis‐Fajdasz et al., 2021), and in the NAD^+^/NADH metabolism (Zhu et al., 2015) during aging.

In this report, we deliver several lines of evidence that inhibition of Pyg alleviated memory deficits and restored neuroplasticity in aged, 20‐ to 22‐month‐old mice. We show that the 2‐week intraperitoneal administration of the Pyg inhibitor BAY U6751 improved memory in old animals, in terms of behavioral skills tested using the 2‐Novel Object Recognition (NOR) and Novel Object Location (NOL) tests, both partially dependent on the hippocampal mechanisms of memory formation.

This reported memory improvement was correlated with significant alterations in the hippocampal formation, at both the cellular and molecular levels, that is, morphological changes in dendritic spines and restoration of young‐like proteome in old animals. At the same time, BAY U6751 did not change performance in—also hippocampus‐contingent—the Y‐maze spontaneous alternations test. This points to the task‐specificity of the BAY U6751‐induced improvement in cognitive function, which is most likely attributable to changes in both hippocampus and in different neural circuits beyond hippocampus involved in the applied cognitive assays.

In contrast to the aged mice, the inhibition of Pyg in 8‐week‐old animals had no effect on overall memory formation as measured by the NOR test; however, it disturbed the hippocampal mechanism of memory formation, changed protein expression, and decreased density of dendritic spines in the hippocampal formation.

Both in young and in aged animals, BAY U6751 did not significantly alter the density and morphology of cortical dendritic spines.

The results presented here demonstrate that inhibition of glycogen breakdown may be a promising target of therapies improving aging‐associated memory decline.

## 2 RESULTS

**FIGURE 1:**
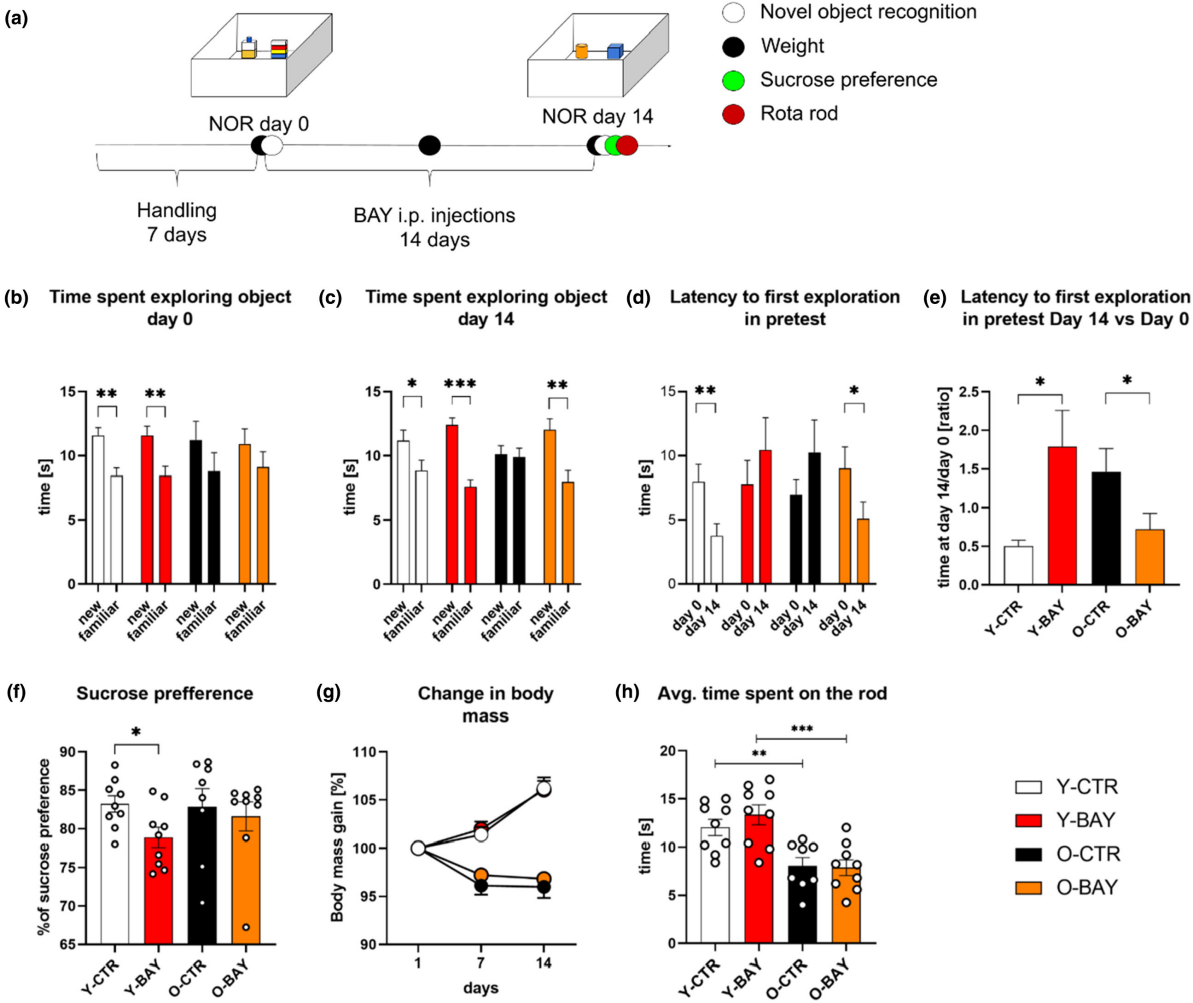
Inhibition of glycogen phosphorylase stimulates memory formation of 20‐ to 22‐month‐old mice. (a) Schematic representation of the experiment; for more details, please refer to the Figure S2a. (b, c) Objects exploration time during the familiarization session of the NOR performed at Day 0 and at Day 14. (d) Latency to the first exploration of any object in the familiarization sessions, and (e) the ratio of the latency to the first exploration between Day 14 and Day 0 as an index of memory formation related to hippocampal plasticity. We observed increased spatial orientation for Y‐CTR and O‐BAY mice (time reduction at Day 14), and decreased spatial orientation for Y‐BAY and O‐CTR mice (time extension at Day 14). (f) Sucrose preference test at Day 14. (g) Body mass index as percentage of body mass at Day 0. (h) Time spent on the rod in the Rotarod test at Day 14. The number of subjects in each experimental group *n* = 9. The statistically significant changes between groups are indicated (*p < 0.05; **p < 0.01; ***p < 0.001).

Previously, we showed that the blockade of glycogen degradation significantly improved the LTP induction in the CA1 region of hippocampal slices dissected from old animals (Drulis‐Fajdasz et al., 2015). Based on these results, we hypothesized that inhibition of Pyg activity would lead to an improvement of age‐associated deficits in memory formation. To verify this hypothesis, we treated young (6‐week‐old) and old (20–22 months old) mice for 2 weeks with daily intraperitoneal injections of the Pyg inhibitor BAY U6751 (BAY; Figure 1a) and tested their behavior, neuronal morphology, and proteome in comparison with control groups of animals injected with saline. The titer of the inhibitor was chosen on the basis of studies using the hippocampal slices of young rats. The lowest titer that completely blocked the LTP formation was selected (Figure S1).

### 2.1 Behavioral studies

The 2‐Novel Object Recognition test (NOR) and Novel Object Location test (NOL) are routinely used to study memory and learning, preference for novelty, the influence of different brain regions on the process of recognition, and to determine effects of drugs on memory formation (Antunes & Biala, 2012; Denninger et al., 2018).The tests are technically simple and do not require aversive or appetitive stimulation, thereby minimizing the animal's stress which could be a confounding factor in cognitive skills measurements. In our experiment, we used the NOR variant without the habituation step and with 6‐h interval between the familiarization and test session. According to Leger et al., this experimental design allows the most sensitive discrimination between the time spent by an animal on exploring the novel object compared to the familiar one (Leger et al., 2013). The NOR test is known to measure multi‐mode brain system interactions in cognition, but it is mainly associated with the cortex and hippocampus functions (Warburton & Brown, 2010). Moreover, omission of the habituation phase enables detection of aspects which are related mainly to hippocampus‐dependent contextual memory formation such as the time to the first exploration of the object (Oliveira et al., 2010).

Data obtained from the NOR test performed at the Day 0 (i.e., before the BAY administration) revealed that, as would be expected, old animals were characterized by impaired novel object recognition compared to young animals. Young mice explored the novel object significantly longer than the familiar one (Y‐CTR *p* = 0.004; Y‐BAY *p* = 0.003), whereas old animals explored the novel and familiar object for a similar length of time (O‐CTR *p* = 0.281; O‐BAY *p* = 0.339) (Figure 1b). We did not find any signs of side preferences in the object exploration (Figure S2).

#### 2.1.1 Pyg inhibition disturbs hippocampal‐dependent object recognition in young mice

After the 2‐week intraperitoneal administration of the phosphorylase glycogen inhibitor (in BAY groups) or saline (in control groups), young animals from both groups continued to spend a significantly higher amount of time on the exploration of the novel object (Y‐CTR *p* = 0.039; Y‐BAY *p* = 1.4 × 10^−5^) (Figure 1c), which suggested that the BAY treatment did not impair the formation of memory about the familiar/constantly present object.

However, in the NOR test with intersession interval (ISI = 6 h), the overall time of exploration of the new object reflects the sum of the cortex‐ and hippocampus‐dependent mechanisms of the long‐term memory formation (Antunes & Biala, 2012). Moreover, Oliveira et al. showed that if familiarization with object occurred when the contextual environment was relatively novel (here: without the habituation phase), the hippocampus played an inhibitory role in the consolidation of object recognition memory. Thus, hippocampal inactivation could even enhance the cortex‐related long‐term memory in young animals (Oliveira et al., 2010).

In the light of these findings, it may be concluded that at Day 14, the Y‐BAY mice expressed abilities similar to the Y‐CTR as a result of the preserved function of cortex and inhibition of the hippocampal plasticity (Figure 1c).

In turn, changes in latency to the first exploration of any object in the NOR test are a measure of the hippocampus‐dependent memory (formed during the familiarization session at Day 0). This parameter is also an indicator of anxiety, as animals displaying elevated anxiety tend to delay exploration of objects (Heinz et al., 2021). Here, we did not observe any differences in the latency between the animal groups in the familiarization session at Day 0 (Figure 1d), which gave us confidence about the homogeneity of the animals in this aspect of behavior.

However, after the second familiarization session at Day 14, we found that the BAY‐treated young animals did not change the latency to the first exploration (*p* = 0.192), whereas in the saline‐treated young mice, the latency was significantly shorter (*p* = 0.01) (Figure 1d,e). Since shortening of time to the first exploration points to improvement in spatial orientation in a familiar environment (Heinz et al., 2021) thus, the lack of such a shortening is an indicator of decreased hippocampal function (disturbance of memory about already seen environment) upon BAY treatment in young animals.

#### 2.1.2 Pyg inhibition improves hippocampal‐dependent cognition in old mice

In contrast to young mice, old animals responded to the BAY treatment with an improved recognition of objects. After 14 days of the treatment, we observed that the novel object recognition index significantly increased, as compared to the baseline level, in the BAY‐injected old mice (O‐BAY Day 0, *p* = 0.339 and O‐BAY Day 14, *p* = 0.004), but not in the saline‐injected group of old animals (O‐CTR Day 0, *p* = 0.282 and O‐CTR Day 14, *p* = 0.423) (Figure 1b,c). The BAY‐injected old mice also showed a significantly shorter time to the first exploration of any object in the familiarization session between the Day 0 and Day 14 of the experiment (*p* = 0.04). This effect was not observed upon saline treatment (*p* = 0.26) (Figure 1d,e). The improved recognition, orientation, and motivation to explore could be considered as an indicator of the increased hippocampal function upon the BAY treatment in old animals (Figure 1d,e).

**FIGURE 2:**
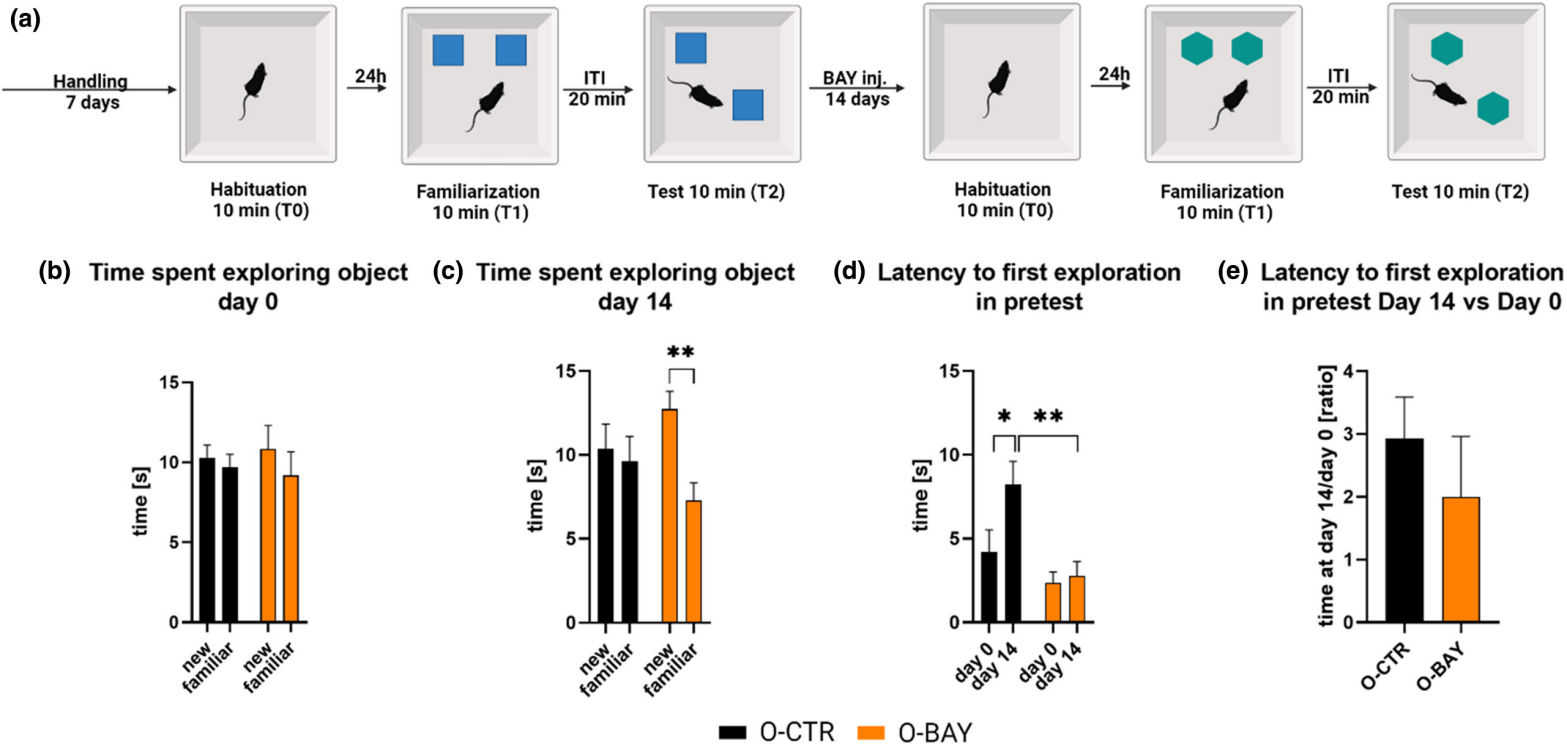
Inhibition of glycogen phosphorylase improves spatial memory in Novel Object Location test (NOL) in 20‐ to 22‐month‐old mice. (a) Schematic representation of the experiment. Exploration time of objects at familiar and new locations during test session (T2) of the NOL performed before (Day 0) and after (Day 14) BAY or saline daily injections (b, c). Latency to the first exploration of any object in the familiarization sessions (d), and the ratio of the latency to the first exploration between Day 14 and Day 0 (e). The number of subjects in each experimental group *n* = 8. The statistically significant changes between groups are indicated (*p < 0.05; **p < 0.01).

Also, the results of the NOL test demonstrated that the prolonged BAY treatment of aged mice improved their spatial memory (Figure 2). We observed that the BAY‐treated old mice explored objects at new locations significantly longer than objects at familiar locations (*p* = 0.0067) (Figure 2c), while at the first day of day of the experiment no such differences were observed (Figure 2b). The BAY treatment had no significant effect on the latency to the first exploration of the objects (Figure 2d,e).

### 2.2 The effect of BAY on blood glucose level, sucrose preference, and motor skills

Since the BAY treatment may have significant effects on the metabolism of glucose, we decided to perform additional tests to control for possible alterations of the baseline physiology and behavior (not associated directly with learning and memory in tested animals) that could introduce bias to our NOR test results. We observed that 2‐week treatment of animals with BAY had no effect on the body mass (Figure 1g) and blood glucose level (with average concentration 8.4 mM) of the animals. Moreover, the treatment did not affect motor skills, ataxia and cerebellum‐dependent coordination, as measured by the Rotarod test. However, as expected, the time which old animals spent on the Rotarod was about 2/3 of that of young animals (Y‐CTR vs. O‐CTR *p* = 0.005; Y‐BAY vs. O‐BAY *p* = 8 × 10^−4^) (Figure 1h). The lack of effects of BAY on locomotor skills in young and old mice may be unexpected; however, we did not use caloric restrictions and did not perform an endurance exercise test which could reveal changes related to the lack of glycogen availability in the tissues.

Since the hippocampus is implicated in the pathophysiology of depression, we decided to check whether the BAY treatment might result in changes of sucrose preference, a parameter used to measure hedonic deficit, a hallmark of depression (Campbell & MacQueen, 2004). The SPT test performed before the BAY treatment revealed no differences between the studied groups (data not shown). We found a slight decrease in sucrose preference in young but not old BAY‐treated animals, compared to the respective saline‐treated group (Y‐CTR vs. Y‐BAY *p* = 0.02; O‐CTR vs. O‐BAY *p* = 0.54) (Figure 1f). Although the decreased synaptic plasticity of the hippocampus upon the BAY treatment may be responsible for this effect, it may as well be attributed to BAY‐related metabolic changes in young organisms. Thus, further studies are necessary to better understand this observation.

### 2.3 The effect of Pyg inhibition and aging on dendritic spines density and morphology

**FIGURE 3:**
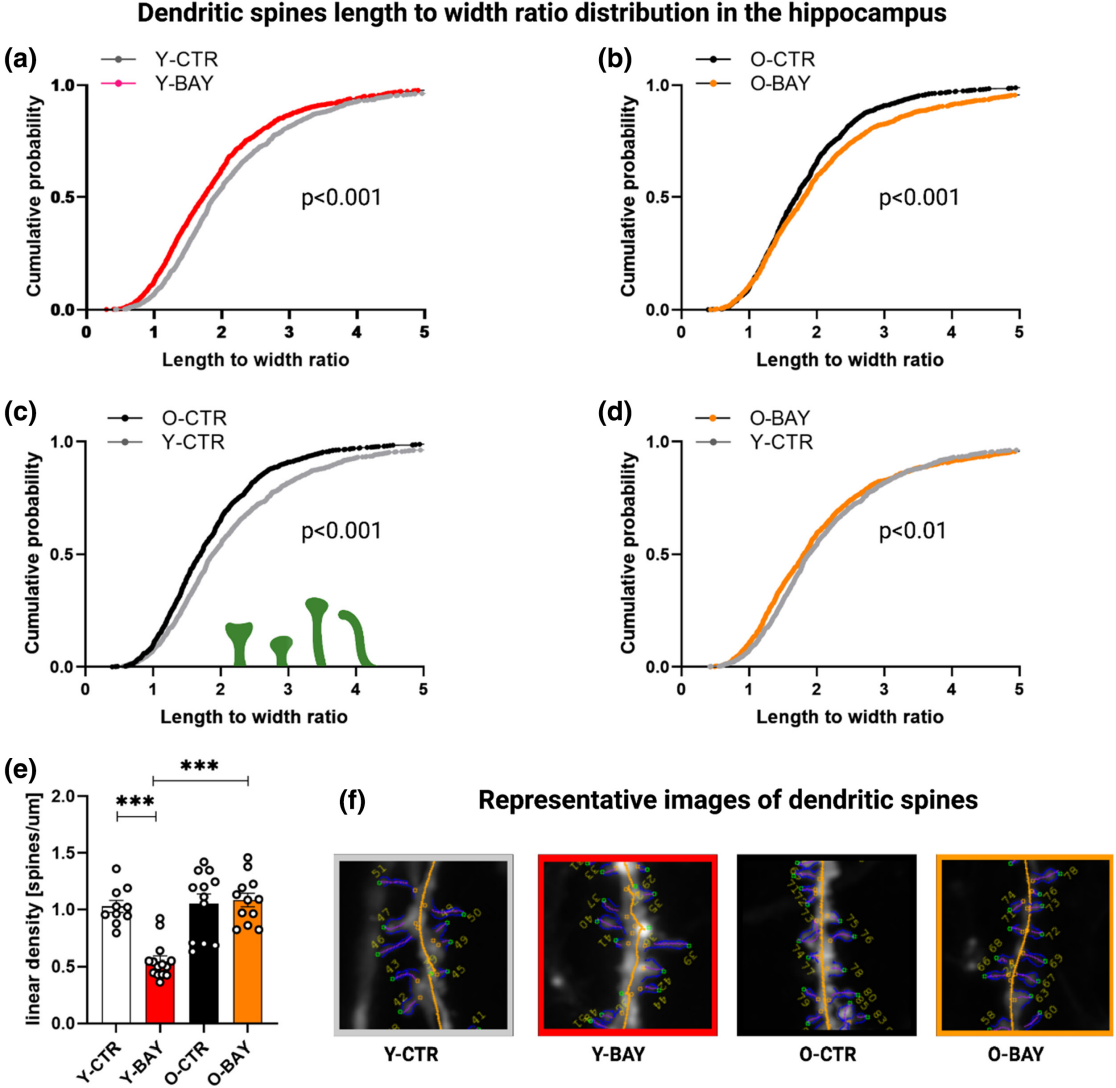
Glycogen phosphorylase inhibitor BAY U6751 affects dendritic spines morphology in hippocampi of young and old animals. (a–d) Distribution of dendritic spines shape parameter (length‐to‐width ratio). (e) Spine linear density in hippocampal neurons. (f) Examples of DiI‐stained neurons in the CA1 area of mouse hippocampus (images show secondary apical dendrites; blue outline—exemplary regions of interest marked for individual dendritic spines; yellow outline—defined dendrite core). The data were analyzed using the Kolmogorov–Smirnov test (a–d) and Student's *t*‐test (e). The number of subjects in each experimental group: animals (*n* = 4–5), cells per animal (*n* = 9–14), and spines per group (*n* = 1100–1250). *** *p* < 0.001.

**FIGURE 4:**
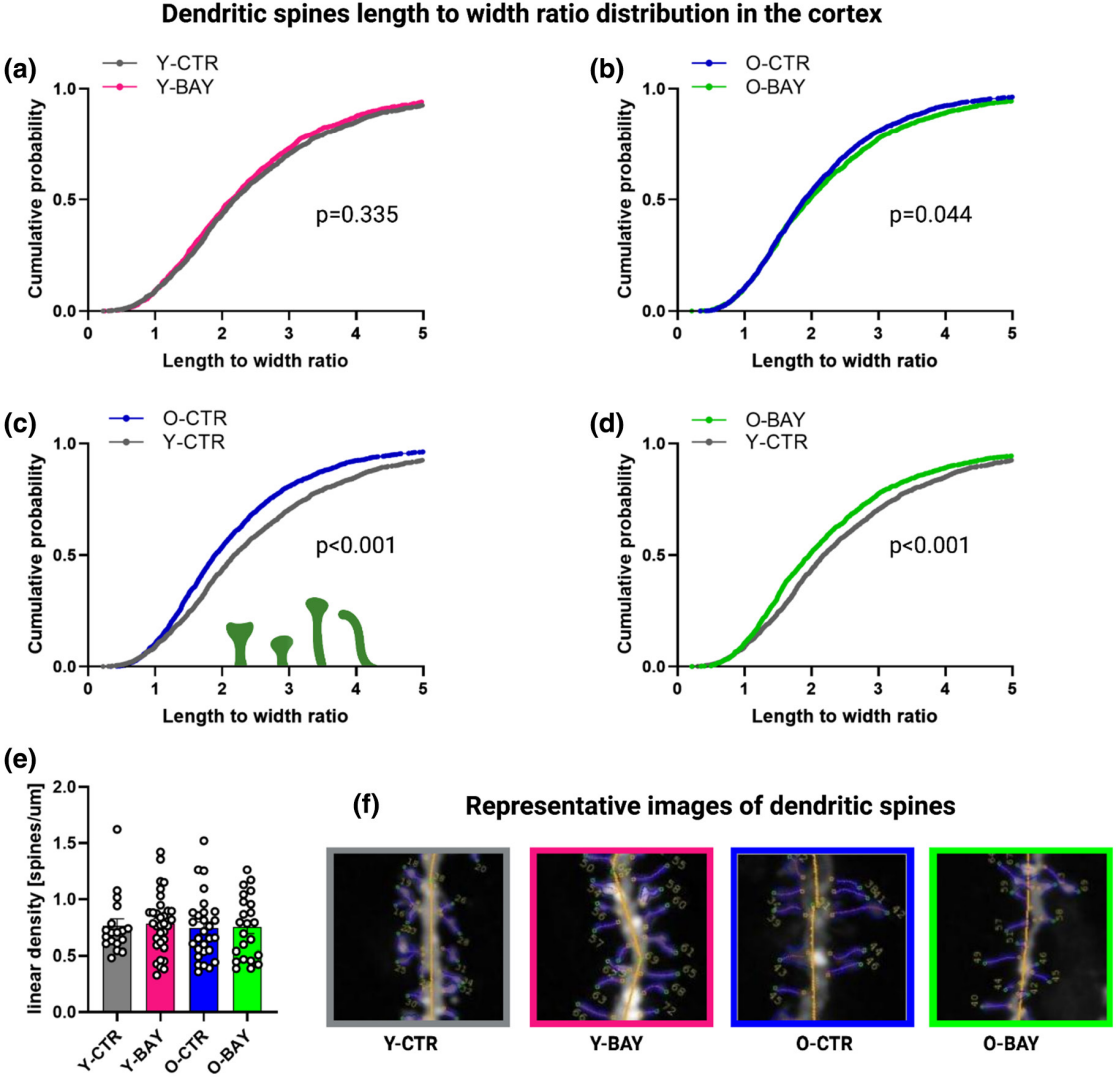
Glycogen phosphorylase inhibitor BAY U6751 do not affect spine morphology in the cortex of young and old animals. (a–d) Distribution of dendritic spines shape parameter (length‐to‐width ratio). (e) Spine linear density column chart. (f) Examples of DiI‐stained neurons in cortex (blue outline—exemplary regions of interest marked for individual dendritic spines; yellow outline—defined dendrite core). The data were analyzed using the Kolmogorov–Smirnov test (a–d) and Student's *t*‐test (e). The number of subjects in each experimental group: animals (*n* = 4–5), cells per animal (*n* = 9–14), and spines per group (*n* = 1100–1250).

In order to check how the Pyg blockade affected neuronal plasticity in young and aged mice, we analyzed morphology and density of dendritic spines in secondary/tertiary dendrites of the CA1 hippocampal region and cortex. Here, we present the scale‐free parameter, the length/width ratio, as cumulative curves to visualize spine distribution according to the spine shape classification—from stubby and mushroom‐shaped, through thin and long thin, to filopodia‐like (Michaluk et al., 2011). The analysis revealed significant differences between young and old groups of untreated mice both in the hippocampus and in the cortex (Figures 3c and 4c).

In agreement with previously published results (Toni et al., 2007), hippocampal and cortical dendritic spines of old mice were enriched in stubby and mushroom spines, as compared to spines of young mice whose dendritic spines were thinner and more filopodia‐like (hippocampus Y‐CTR vs. O‐CTR: *D* = 0.128, ks = 3.1, *p* = 1 × 10^−6^; cortex Y‐CTR vs. O‐CTR: *D* = 0.117, ks = 3.23, *p* = 1 × 10^−6^) (Figures 3c and 4c).

Two weeks of BAY administration resulted in changes of the hippocampal dendritic spine morphology both in young and in aged animals, compared to their respective control groups.

In young animals, Pyg inhibition was associated with the shift of the CA1 hippocampal spines morphology toward shorter and thicker spines (Y‐CTR vs. Y‐BAY: *D* = 0.11, ks = 2.46, *p* = 1 × 10^−6^) (Figure 3a), that are associated with more stable synapses formed upon LTP induction (Szepesi et al., 2014). These morphological changes in response to the BAY treatment were, however, accompanied by a significant decrease in the spine density, compared to young control group (Y‐CTR = 1.02 spines/μm; Y‐BAY = 0.54 spines/μm; *p* = 6.6 × 10^−6^) (Figure 3e) which may suggest that the Pyg activity blockade caused elimination of immature (long and thin) spines rather than spine maturation.

In contrast, in old animals, we did not find significant changes in the spine density of the CA1 hippocampal dendrites upon the BAY treatment (O‐CTR = 1.05 spines/μm vs. O‐BAY = 1.08 spines/μm: *p* = 0.79) (Figure 3e). Moreover, the spine morphology analysis revealed that the prevalence of mature spines remained on similar levels in old control and BAY‐treated mice (O‐CTR vs. O‐BAY within spines with the length‐to‐width ratio below 2: *D* = 0.038; ks = 0.76; *p* = 0.614) (Figure 3b). However, when we looked into the immature spine compartment (spines with the length‐to‐width ratio above 2), we found that the BAY treatment resulted elongation and thinning of the spines, that is, the spines became more filopodia‐like (O‐CTR vs. O‐BAY: *D* = 0.166, ks = 2.5, *p* = 7 × 10^−6^) (Figure 3b). Since the abundance of the filopodia‐like spines is associated with increased potential for new synapse formation thus, the improvement of memory formation of BAY‐treated aged mice may be at least partially attributed to the improved synaptic plasticity.

The O‐BAY group's changes in hippocampal dendritic spines were analogical (but more prominent) to that observed by us previously for rat acute hippocampal slices treated with the glycogen phosphorylase inhibitor ex vivo (Drulis‐Fajdasz et al., 2015). Remodeling of the dendritic spines shape is a marker of structural plasticity within hippocampus excitatory synapses, and this plasticity is probably responsible for the enhanced memory formation observed in old rats and mice.

Unexpectedly, in the cortex, neither dendritic spine densities (Y‐CTR vs. Y‐BAY *p* = 0.786; O‐CTR vs. O‐BAY *p* = 0.948) (Figure 4e) nor morphology of spines were significantly affected by the BAY treatment (Y‐CTR vs. Y‐BAY: *D* = 0.036, ks = 0.942, *p* = 0.337 and O‐CTR vs. O‐BAY: *D* = 0.047, ks = 1.37, *p* = 0.045) (Figure 4a,b). Also, the statistically significant basic differences between young and old control groups that were present in cortex (Y‐CTR vs. O‐CTR: *D* = 0.118, ks = 3.23, *p* = 1 × 10^−6^) (Figure 4c) remained unchanged by the BAY treatment (Y‐CTR vs. O‐BAY: *D* = 0.099, ks = 2.73, *p* = 1 × 10^−6^) (Figure 4d).

These outcomes partially explain the results of the NOR test for young mice (Figure 1c). After 14 days of the BAY treatment, the mice preserved learning abilities despite the apparent elimination of immature hippocampal dendritic spines (Figure 3a,e). On the contrary, since our experiments were conducted between 6 and 8 weeks of age (with the final NOR test performed on 8‐week‐old mice) thus, the high physiological lactate concentration in the brain (Chen et al., 2016), along with intensive neurodevelopmental processes that occur in such young animals (Chen et al., 2018), may be sufficient to compensate for the impairments caused by the BAY inhibitor in hippocampi.

### 2.4 Inhibition of glycogen phosphorylase alters global hippocampal proteome of young and old mice

**FIGURE 5:**
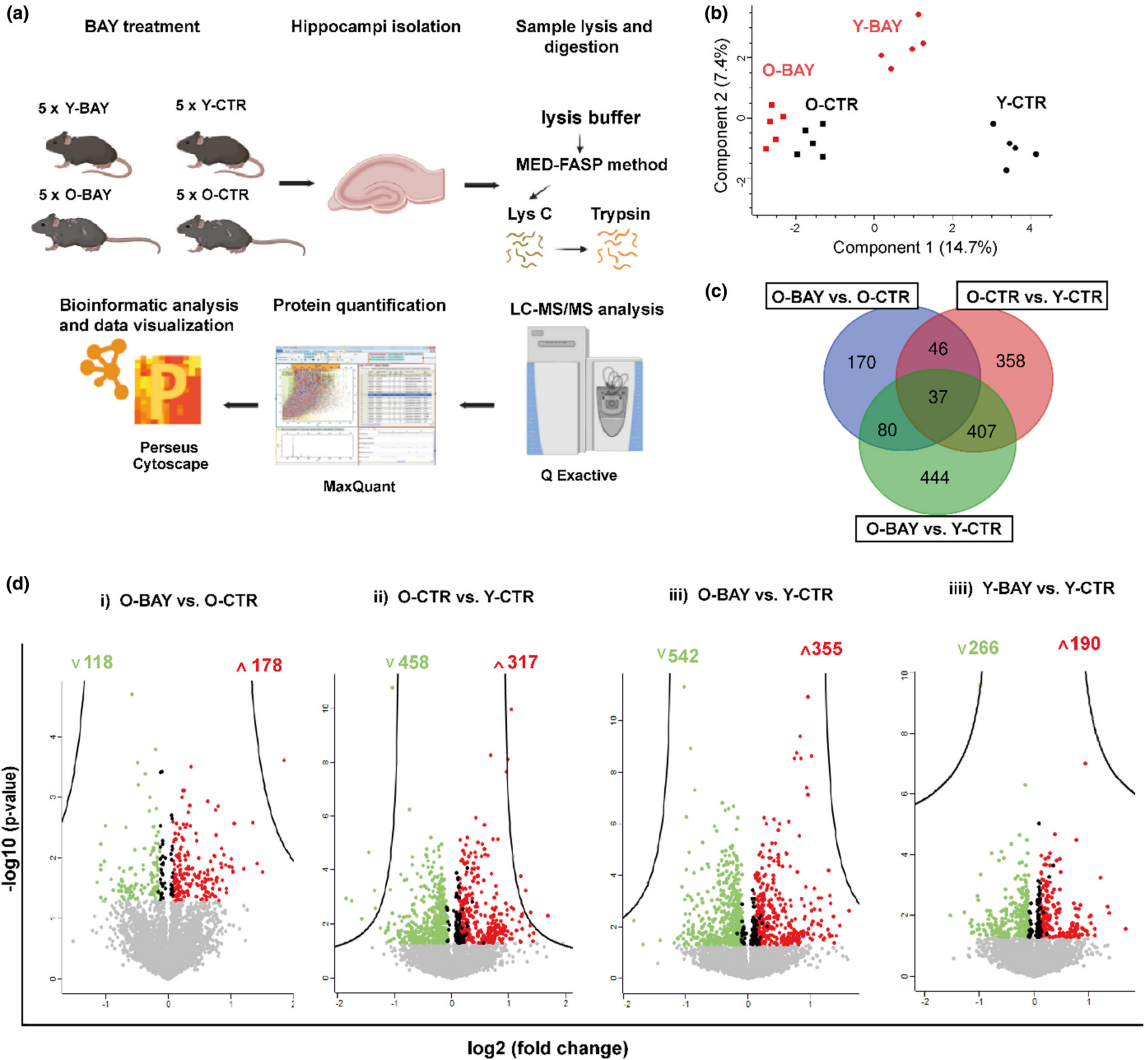
Protein abundance in old and young mice after the BAY treatment. (a) Experimental overview for LC–MS/MS analysis of the hippocampi in 8‐week‐old and 20‐ to 22‐month‐old mice (*n* = 5 biological replicates per group). (b) Principal component analysis (PCA) of log2 intensity values from all experimental groups. PCA was performed with the use of the Perseus software (Max Planck Institute for Biochemistry, Martinsried, Germany). (c) Proteins with significantly changed abundance and with a fold change ≥1.2 overlapping experimental groups. (d) Volcano plots comparing the protein abundances in (i) BAY treated old mice versus old controls, (ii) old controls versus young controls, (iii) BAY treated old mice versus young controls, (iiii) BAY treated young mice versus young controls. Red and green circles represent significantly up‐ and down‐regulated proteins, respectively, with a fold change ≥1.2. Black circles represent all other significantly changed proteins. Gray circles display proteins not regulated by BAY/age. The number of upregulated (red) and down‐regulated (green) proteins is presented. Proteins were graphed by fold change (difference) and significance (−log *p*‐value) using a false discovery rate (FDR) of 0.05, with the Perseus software.

The overview of the proteomic experiment is presented in Figure 5a. A total of 7959 proteins with at least two unique peptides were identified across all study samples. The mass spectrometry proteomics data have been deposited to the Proteome Xchange Consortium via the PRIDE partner repository with the dataset identifier PXD025978, file UU6‐UU15.

**FIGURE 6:**
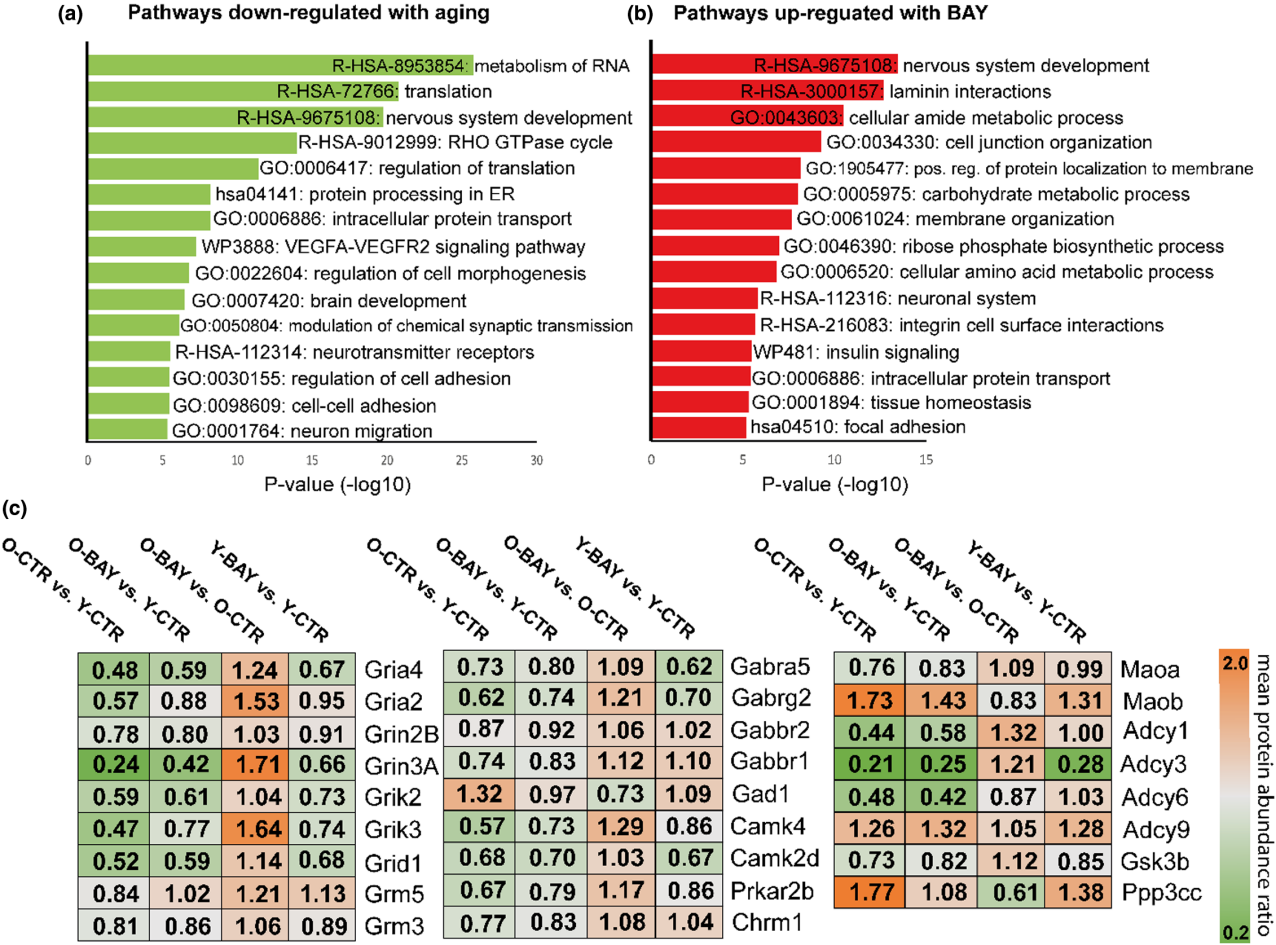
Metascape enrichment analysis of (a) proteins down‐regulated with age (O‐CTR vs. Y‐CTR), and (b) proteins upregulated by the BAY treatment of old mice (O‐BAY vs. O‐CTR). Enrichment *p*‐values were generated by the Metascape using cumulative hypergeometric distributions. GO—Gene Ontology, R‐HSA—Reactome pathway database, WP—WikiPathways. (c) Changes in the abundance of the proteins involved in synaptic transmission and neuroplasticity across studied groups. The values represent the ratio of the mean abundance between studied groups (O‐CTR vs. Y‐CTR, O‐BAY vs. Y‐CTR, O‐BAY vs. O‐CTR, Y‐BAY vs. Y‐CTR). The value is then assigned colors according to the scale on the right.

For quantitative analyses, only proteins identified in at least 60% of samples belonging to a given study group were used (6147 proteins in total). The principal component analysis (PCA) of protein concentrations exhibited changes in each analyzed proteome related to age and BAY treatment (Figure 5b). We observed that inhibition of glycogen phosphorylase significantly affected the abundance of several proteins in old mice (340 proteins in total, *p* < 0.05), 178 and 118 of which were up‐ and down‐regulated, respectively (please see the Table S1). In the Figure 5c, the differentially abundant proteins (DAPs) overlapping three experimental groups (O‐BAY vs. O‐CTR; O‐CTR vs. Y‐CTR, and O‐BAY vs. Y‐CTR) are shown. All proteins are listed in the Table S2. Finally, the volcano plot visualizes DAPs related to BAY treatment and age (Figure 5d). Interestingly, the hippocampal proteome of the BAY‐treated old animals was more divergent from the proteome of young control mice (Figure 5d iii) than was the proteome of the old control mice (Figure 5d ii) which might suggest that BAY induced some cognitive anomalies in old animals. However, a more detailed analysis of protein changes presented in Figure 6 showed that titers of proteins crucial for neuronal transmission and neuroplasticity in old animals were restored by BAY treatment to young‐like concentrations.

Similarly to previous studies, we found that many fundamental pathways were down‐regulated with aging, including translation, nervous system development, protein transport, cell adhesion and migration, and neurotransmission (Figure 6a). In turn, enrichment analysis of BAY‐related proteins with increased abundance revealed a significant up‐regulation of many of the above processes suggesting an overall shift of the aged hippocampal proteome toward the young‐like proteome (Figure 6b). Notably, in BAY‐treated old mice, we observed changes in the abundance of several proteins involved in neuronal transmission and memory formation toward young‐like proteome. Among these proteins were key excitatory glutamate receptors (both the ionotropic, such as Gria, Grin, Grik, Grid, and the metabotropic, such as Grm) (Figure 6c). The BAY treatment elevated levels of receptors for the main inhibitory neurotransmitter in the hippocampus—gamma‐aminobutyric acid (GABA): ionotropic (Gabr) and G‐protein coupled (Gabbr) receptors. It also upregulated the level of muscarinic acetylcholine receptor (Chrm) (Figure 6c). In line with the above “rejuvenating” tendency, we found changes in expression of several protein kinases (Camk, Prkar, Adrbk, and GSK3b), protein phosphatases (Ppp3cc), adenylate cyclases (Adcy), and proteins involved directly in neurotransmitter metabolism (Mao) (Figure 6c).

**FIGURE 7:**
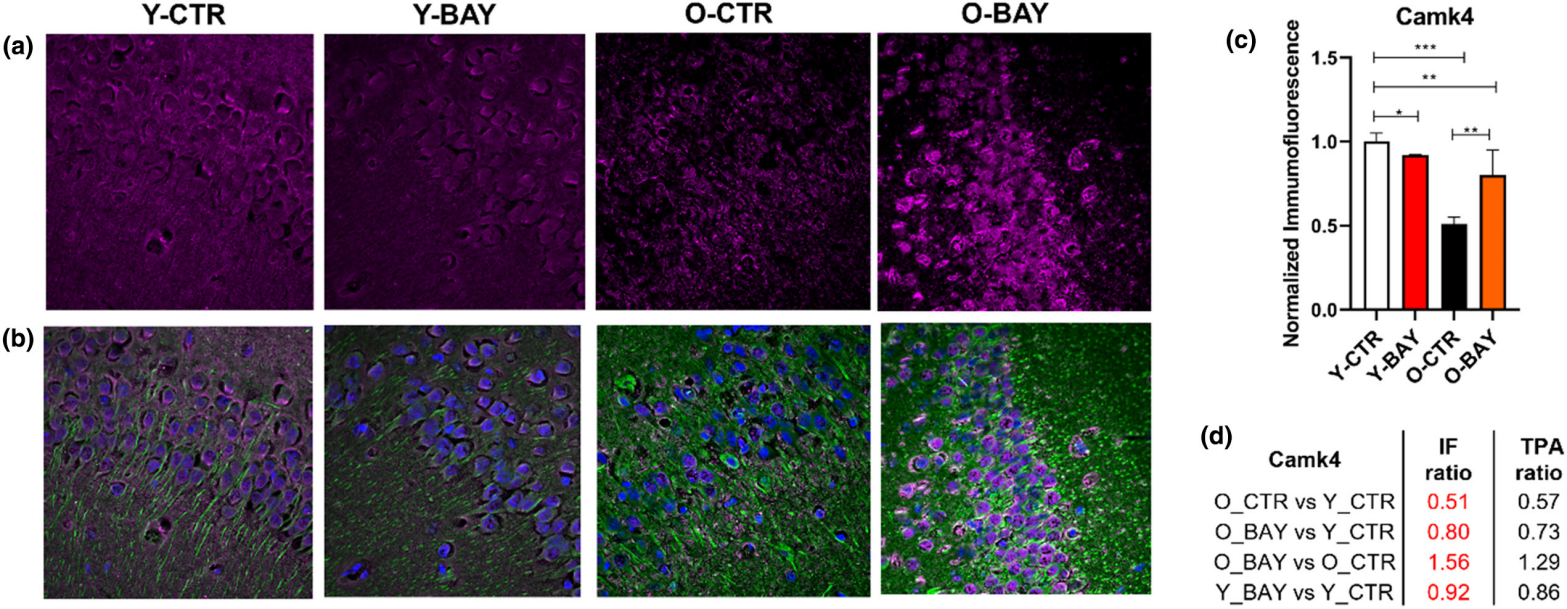
Calcium/calmodulin‐dependent protein kinase type IV (Camk4) expression in the hippocampus is altered by aging and BAY treatment. (a) Exemplary confocal images of Camk4 immunofluorescence (magenta) distribution within hippocampal *Cornu Ammonis* (CA1) stratum pyramidale (SP) region in young (Y‐CTR), young BAY treated (Y‐BAY), old (O‐CTR) and old BAY treated (O‐BAY) animals, respectively. (b) Localization of neuronal somata, dendrites, and nuclei was revealed with antibodies against β‐Tubulin Isotype III (green) and DAPI (blue), respectively (merge channels). (c) Quantification of Camk4‐related immunofluorescence in hippocampal slices calculated for total hippocampi CA1 and CA2 region (SP, stratum pyramidale; SR, stratum radiatum; stratum lacunosum‐moleculare, SLM). Immunofluorescence was normalized to values obtained for Y‐CTR samples (*n* = 6). Asterisks indicate a statistically significant difference (*p < 0.05, **p < 0.01, ***p < 0.001). (d) Relative immunofluorescence ratio between analyzed animal groups (IF ratio) in comparison with protein concentration ration obtained in proteomic analysis (TPA ratio).

**FIGURE 8:**
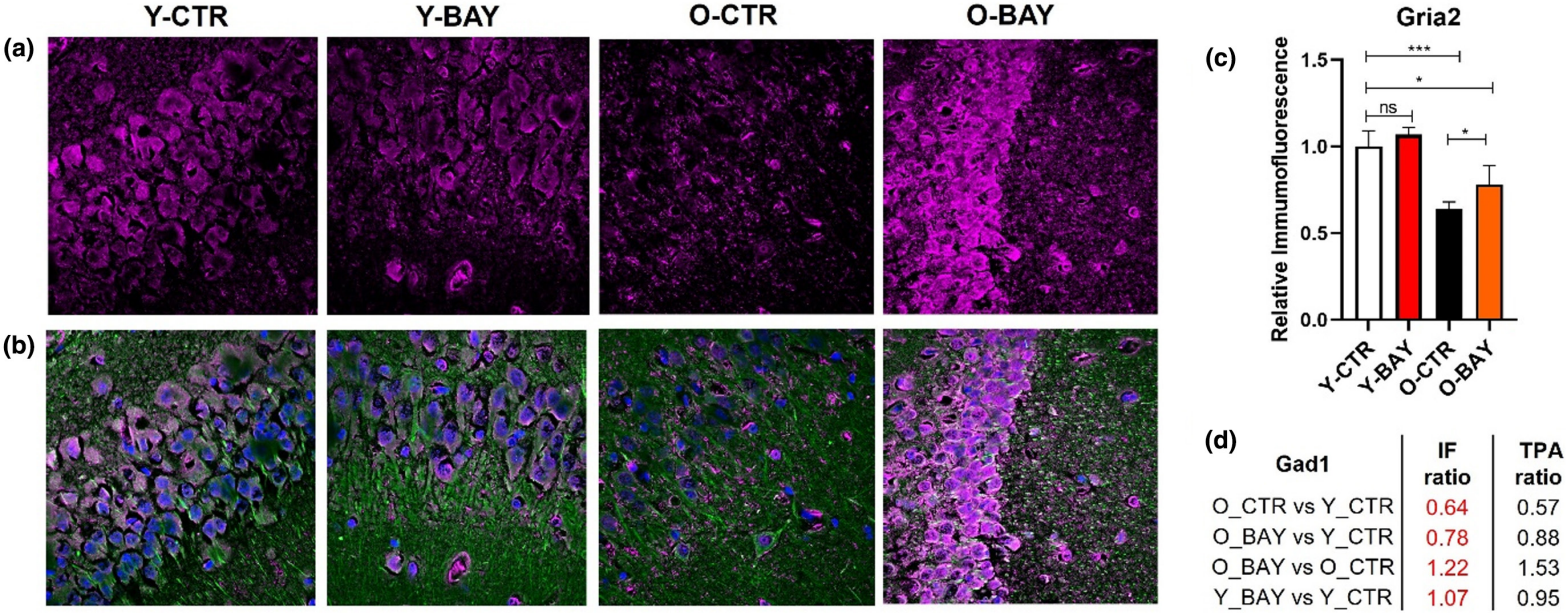
Cellular localization of Glutamate receptor 2 (Gria2 = GluR2) in the hippocampus is altered with aging and is altered with BAY inhibitor treatment. (a) Exemplary confocal images of Gria2 immunofluorescence (magenta) distribution within hippocampal *Cornu Ammonis* (CA1) stratum pyramidale (SP) region in young (Y‐CTR), young BAY treated (Y‐BAY) old (O‐CTR) and old BAY treated (O‐BAY) animals respectively. (b) Localization of neuronal somata, dendrites, and nuclei as revealed with antibodies against β‐Tubulin Isotype III (green) and DAPI (blue), respectively (merge channels). (c) Quantification of Gria2 normalized immunofluorescence in hippocampal slices calculated for total hippocampi CA1 and CA2 region (SP, stratum pyramidale; SR, stratum radiatum; stratum lacunosum‐moleculare, SLM). Immunofluorescence was normalized to values obtained for Y‐CTR samples (*n* = 6). Asterisks indicate a statistically significant difference (*p < 0.05, **p < 0.01, ***p < 0.001). (d) Relative immunofluorescence ratio between analyzed animal groups (IF ratio) in comparison with protein concentration ration obtained in proteomic analysis (TPA ratio).

**FIGURE 9:**
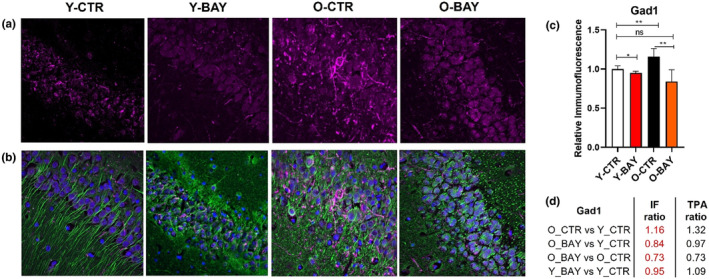
Cellular localization of Glutamate decarboxylase 1 (Gad1 = Gad67) in the hippocampus is altered with aging and is altered with BAY inhibitor treatment. (a) Exemplary confocal images of Gad1 immunofluorescence (magenta) distribution within hippocampal *Cornu Ammonis* (CA1) stratum pyramidale (SP) region in young (Y‐CTR), young BAY treated (Y‐BAY) old (O‐CTR), and old BAY treated (O‐BAY) animals respectively. (b) Localization of neuronal somata, dendrites, and nuclei as revealed with antibodies against β‐Tubulin Isotype III (green) and DAPI (blue), respectively (merge channels). (c) Quantification of Gad1 normalized immunofluorescence in hippocampal slices calculated for total hippocampi CA1 and CA2 region (SP, stratum pyramidale; SR, stratum radiatum; stratum lacunosum‐moleculare, SLM). Immunofluorescence was normalized to values obtained for Y‐CTR samples (*n* = 6). Asterisks indicate a statistically significant difference (*p < 0.05, **p < 0.01, ***p < 0.001). (d) Relative immunofluorescence ratio between analyzed animal groups (IF ratio) in comparison with protein concentration ration obtained in proteomic analysis (TPA ratio).

To validate the MS‐based results, we performed immunofluorescent (IF) labeling of hippocampal slices with antibodies directed against: calcium/calmodulin‐dependent protein kinase type IV (Camk4), glutamate receptor 2 (Gria2 = GluR2) and glutamate decarboxylase 1 (Gad1 = Gad67) (Figures 7, 8, 9). The intensity of the IF staining confirmed that concentrations of the selected proteins changed significantly with aging. Moreover, the titers of the studied proteins were modulated differently by BAY in young and old animals (Figures 7a–c, 8a–c and 9a–c). In old animals, the protein concentrations of Camk4 and Gria2 significantly increased, but the concentration of Gad1 significantly decreased. The changes in young animals were opposite and less significant than in old animals: The Camk4 and Gria2 concentrations decreased, while the Gad1 protein level increased. Together, the BAY treatment reduced the age‐related differences in protein levels.

Comparing the changes in fluorescence intensities related to the studied proteins between all analyzed groups (Y‐CTR, Y‐BAY, O‐CTR, and O‐BAY), we found a direct correlation with the changes in proteins concentrations measured by the proteomic analysis (TPA ratio) (Figures 6c, 7d, 8d and 9d).

### 2.5 LC–MS quantification of the BAY in mouse hippocampi and cortex

We used the LC–MS method to identify and quantify BAY U6751 in brain structures, to confirm that intraperitoneal administration of the inhibitor allowed to achieve effective working concentrations in the hippocampus and cortex. Measurements were made based on three technical repetitions, and obtained concentration values are above the limit of quantification (LOQ) in the developed method (≥23.29 ng/mL).

We found that the average concentration for BAY in the hippocampus and cortex tissue extracts at Day 14 was 24.42 ± 3.64 ng/mL and 57.13 ± 3.13 ng/mL, respectively. The measurement precision was expressed as the coefficient of variation (CV) and did not exceed ±15% (for hippocampal = 14.92%; for cortex = 5.5%; see Methods 5.14). Representative extracted ion chromatograms for BAY in hippocampus and in cortex are presented in Figure S5.

The LC–MS measurements confirmed that BAY effectively penetrated the tissues and crossed the blood–brain barrier. The measured concentrations of the compound were similar to concentrations determined in our previous study: 23.66 ± 0.96 ng/mL (CV = 4.07%) in the hippocampus and 58.92 ± 1.21 ng/mL (CV = 2.05%) in the cortex (Pudelko‐Malik et al., 2022).

## 3 DISCUSSION

Memory loss and cognitive impairments are observed in numerous neurodegenerative diseases. However, a gradual decrease in cognitive functions such as memory formation and its retention also occurs naturally as a part of the aging process in individuals with no evident symptoms of pathological neurodegeneration (Harada et al., 2013). Endurance exercises and healthy lifestyle are known and commonly accepted factors which delay brain aging and preserve memory and cognition (for review see in Sujkowski et al., 2022). Recently, it was suggested that several pharmacological compounds such as senolytics (Krzystyniak et al., 2022; Ogrodnik et al., 2021) and inhibitors of the integrated stress response may contribute to the improvement of age‐related cognitive (Chou et al., 2017; Krukowski et al., 2020; Oliveira et al., 2021). Our previous studies on a molecular/cellular mechanism of memory formation—LTP, and aging‐dependent alterations in hippocampal proteomes suggested that glycogen metabolism may be a promising target of an anti‐aging brain therapy.

It is widely accepted that the astrocytic glycogen‐derived lactate is indispensable for the hippocampus‐associated memory formation in young individuals (Gibbs et al., 2006; Newman et al., 2011; Suzuki et al., 2011). The mechanism of the lactate‐dependent stimulation of the LTP formation is not fully understood but it is hypothesized that the crucial role in this process plays an increasing NADH/NAD^+^ ratio in neurons, which is a result of astrocytic glycogen‐derived lactate oxidation (Magistretti & Allaman, 2015). The elevation of the NADH/NAD^+^ ratio may lead to potentiation of NMDA signaling (Yang et al., 2014) and promotion of Camk2 autoactivation by dimeric fructose‐1,6‐bisphosphatase 2 (Fbp2) (Duda et al., 2020), an enzyme whose oligomerization (tetramerization) is NAD^+^‐ and AMP‐dependent (Gizak et al., 2019).

In contrast to young animals, in hippocampal slices isolated from old rodents, the inhibition of glycogen degradation significantly elevated the amplitude of the LTP (Drulis‐Fajdasz et al., 2015). Thus, the main aim of the present study was to investigate whether a prolonged inhibition of glycogen phosphorolysis could modify old mice behavior.

The induction and maintenance of the LTP trigger persistent changes in gene expression. It was shown that the LTP induction correlated with an increase in the expression of more than 350 genes, while the level of transcripts for about 240 genes was lowered (Chen et al., 2017). Therefore, we also hypothesized that a prolonged downregulation of Pyg activity may prevent the aging‐related cognitive decline by modification of hippocampal proteome and remodeling neuronal morphology. In contrast to previous studies (of our and other groups) in which changes of memory formation were monitored after a short‐term treatment with Pyg inhibitors (Drulis‐Fajdasz et al., 2015; Gibbs et al., 2006; Kilic et al., 2018), in the present work, we applied the 14‐day protocol of daily injections of mice with the inhibitor (Figure 1a). We found that this treatment did not affect basic physiological parameters of young and old animals such as body mass, serum glucose level, and motor coordination. On the contrary, the consumption of sucrose by young animals treated with BAY was slightly but statistically significantly reduced, which indicated a tendency to the anhedonic‐like behavior of that group of animals and might suggest alterations in the mesocortical limbic system activity (Campbell & MacQueen, 2004). Interestingly, we did not observe significant alterations in glycogen and lactate levels in hippocampi of the BAY‐treated old mice compared to the untreated animals (Figure S6a,b). However, these findings are consistent with results of studies on another glycogen phosphorylase inhibitor, 1,4‐dideoxy‐1,4‐imino‐D‐arabinitol (DAB). They demonstrated that DAB had an inhibitory effect also on glycogen synthase and that prolonged exposure to the inhibitor reduced glycogen levels (Walls et al., 2008). BAY is supposed to have a similar secondary effect (Latsis et al., 2002). Thus, after the long‐term treatment with BAY only moderate changes in glycogen and glycogen‐derived lactate could be expected.

The results of the NOR and NOL tests revealed that the 2‐week treatment with BAY significantly improved spatial orientation and memory formation in old mice. The parameters of the NOR test that are related to both the cortical and hippocampal components of memory formation were moderately changed only in the BAY‐treated old mice (Figure 1c). However, after the BAY‐treatment, the parameters reflecting mainly the hippocampus‐based mechanisms of memory formation were significantly improved in old (Figures 1d,e and 2c), but worsened in young animals (Figure 1d,e).

Intriguingly, it turned out that in contrast to the BAY‐induced cognitive improvement observed in the NOR and NOL tests, the Y‐maze test (Figure S3a) showed no differences between the BAY‐treated and untreated old animals in working memory (Figure S3b–e).

We argue that such effects might be attributable to the at least partially different neural circuits underlying the diverse cognitive functions measured in NOR, NOL, and Y‐maze assays. Indeed, the said circuits are partially located in the structures, which were not the scope of this study.

It is noteworthy, the BAY delivery in our experiments was systemic rather than targeted to a specific brain region. Hence, we are unable to unequivocally attribute the results of the testing for specific cognitive functions to the improvement of plasticity in hippocampus alone. For example, it is feasible that the NOL and NOR‐related hippocampal processing of the input from the parahippocampal, and perirhinal/entorhinal cortex might be differently influenced by the drug than the heavily hippocampus‐dependent spatial working and reference memory, tested in the Y‐maze. Nonetheless, the observed changes in morphology and density of dendritic spines (Figures 4 and 5) point to the hippocampus as the main formation affected by the long‐term BAY treatment.

On the contrary, the prolonged BAY‐treatment influenced electrophysiological recordings in the acute hippocampal slices post Y‐maze behavioral training. While the basal transmission (the amplitude of the AMPAR‐mediated field excitatory postsynaptic potentials normalized to the fiber volley amplitude) was not affected in the BAY‐treated group compared to the control group (Figure S4a–c), the LTP in the BAY‐treated aged animals after Y‐maze learning paradigm was reduced (Figure S4d–f). However, this unexpected observation is consistent with previous reports showing that sometimes it is not feasible to evoke the high‐frequency stimulation‐induced hippocampal LTP after exposition of animals to a new context or training, due to the process called LTP‐occlusion (Li et al., 2005; Whitlock et al., 2006). We argue that due to the BAY‐enhanced receptiveness of the neural circuits of the aged mice to change, plasticity saturation happened more readily than in the untreated controls, which led to the observed effects.

However, to deepen our understanding of the correlation between the BAY‐induced enhancement of cognitive functions and synaptic plasticity, similar electrophysiological experiments should be performed before and after the NOR and NOL tests.

Taken together, our results showed (a) the task‐specificity of the BAY‐induced improvement in cognitive functions, which is most likely attributable to changes in both hippocampus and in different neural circuits beyond hippocampus involved in the specific behaviors tested, and (b) a complex and most probably non‐linear relationship between the BAY treatment and synaptic plasticity induced by training.

The worsening of the hippocampus‐based memory was observed by Magistretti's and Gibbs's groups which showed that inhibition of glycogen degradation and lactate release from astrocytes in young animals, impaired their hippocampus‐based memory formation (Gibbs et al., 2006; Suzuki et al., 2011; Vezzoli et al., 2020). The results presented here showed that the 2‐week inhibition of Pyg in young mice weakened their memory to the level observed in old animals, whereas it improved the memory of old mice to the level typical for young animals (Figure 1c). The molecular mechanism of such opposite effects of Pyg inhibition in young and old animals is enigmatic and requires further studies. Pyg activity in the brain is attributed almost exclusively to astrocytes (Newman et al., 2011), and the role of the enzyme in neurons is controversial. Some literature data suggested that Pyg activity in neurons, although low, had a physiological significance (Saez et al., 2014) but other studies showed that the activity of neuronal Pyg was permanently blocked by phosphorylation (Vilchez et al., 2007).

The brain aging was shown to correlate with an increasing acidification, elevation of lactate titer (Ross et al., 2010), mild upregulation of the overall glycolytic enzymes concentration (Drulis‐Fajdasz et al., 2018; Gostomska‐Pampuch et al., 2021), and an increase of the NADH/NAD^+^ ratio (Zhu et al., 2015). It was also shown that supraphysiological concentrations of lactate in young mouse brains could mimic disturbances observed in the old brain (Das et al., 2022). Thus, an attractive hypothesis is that the inhibition of glycogen degradation in old brains may reduce the permanently elevated lactate level and neutralize acidification thereby improving the ability to form the LTP.

Our observation that Pyg inhibition affected mainly hippocampal mechanisms of memory formation prompted us further to check the density and morphology of dendritic spines in both brain formations. We found that BAY reduced the density of dendritic spines (Figure 3e) and significantly shortened their length in hippocampi of young mice (Figure 3a). A reduction of these parameters is considered as a measure of lower plasticity and is related to a loss of spatial orientation and increase of an anhedonic behavior (Krzystyniak et al., 2019). On the contrary, in old animals, the BAY treatment led to a significant shift of dendritic spines population from the stubby and mushroom‐like shapes (that are considered to form stable synaptic connections) (Toni et al., 2007), toward the long and thin, and filopodia‐like structures (Figure 3b). Such filopodia‐like dendritic spines are the main form of spines in young animals and are considered as a hallmark of high neuronal plasticity (Basu et al., 2016). In contrast to hippocampal formation, we did not observe any significant alterations in the density and morphology of cortical dendritic spines either in young or in old BAY‐treated animals (Figure 4), which was consistent with our observation that inhibition of Pyg affected predominantly the hippocampus‐based memory. Because the effect of BAY on memory formation is related to a disturbance in the synthesis of lactate from astrocytic glycogen thus, the lack of influence of Pyg inhibition on cortex‐based mechanisms of memory formation and cortical dendritic spines suggests that the ANLS is essential for memory formation in the hippocampal formation, but in the cortex, the ANLS, although functional (Voutsinos‐Porche et al., 2003), plays only a supplementary role. The observed significant alterations of dendritic spines prompted us to test whether the prolonged Pyg inhibition was reflected by changes in levels of hippocampal proteins. Our previous studies showed that aging correlated with global alterations in the hippocampal proteome (Drulis‐Fajdasz et al., 2021; Gostomska‐Pampuch et al., 2021). Here, we demonstrated that the 2‐week treatment with BAY changed concentrations of hundreds of hippocampal proteins (Figure 5b–d). The detailed proteomic analysis revealed BAY‐induced overall rejuvenation of the old hippocampal proteome (Figures 5 and 6). While in the control animals, aging was correlated with downregulation of protein groups associated with translation, nervous system development, protein transport, cell adhesion and migration, and neurotransmission, the BAY treatment of aged animals increased back the abundance of proteins engaged in many of these processes. Although we cannot definitely exclude that BAY directly affected gene expression by interactions with DNA regulatory sequences, the differences in BAY‐induced changes between young and old hippocampal proteomes point to Pyg‐inhibition‐associated changes in metabolites as the trigger of proteomic remodeling.

## 4 CONCLUSIONS

Our study presents a new insight into the role of glycogen in the aging brain. In contrast to young animals, inhibition of glycogen degradation improves memory formation in old animals, affecting mainly functions of the hippocampal formation.

Therefore, our findings provide direct evidence that inhibition of glycogen phosphorolysis improves memory formation in old animals, and thus, they can contribute to development of better treatment strategies for aging‐related memory deficits. However, detailed studies on the long‐term effects of Pyg inhibitor(s)—also administered through alternative routes (oral or intravenous)—on cognitive functions and physiological parameters of animals in different age, are needed to evaluate the therapeutic potential of the glycogen metabolism inhibition.

## 5 METHODS

### 5.1 Animals

Female C57BL/6J mice, 5 weeks old (juvenile—further referred to as young [Y]; *n* = 18) and 20–22 months old (further referred to as old [O]; *n* = 46), were obtained from the Hirszfeld Institute of Immunology and Experimental Therapy PAN, Wroclaw, Poland, and housed in pairs under the 12 h/12 h light/dark cycle (lights on at 4:00 AM), with food and water available ad libitum. All experiments were carried out in accordance with the Polish guidelines and regulations regarding the care and the use of animals for experimental procedures, and approved by the Wroclaw Ethical Committee (permission no. 10/2018 and no. 041/2023). All efforts were made to minimize the number of animals used in the experiments and to limit their distress and suffering.

### 5.2 Behavioral tests

To determine the impact of glycogen phosphorylase inhibition on cognitive skills of the mice, all animals were subjected to two rounds of the 2‐Novel Object Recognition test (NOR; please see Methods 5.3) and Novel Object Location Test (NOL, please see Methods 5.4), separated by a pharmacological intervention. Before the start of the test (NOR and NOL), the mice were handled for 5 min daily for 1 week to accustom them to the experimenter's touch (Figures 1a and 2a). The first round of the NOR and NOL was performed in Day 0 (before a pharmacological treatment) in order to determine baseline cognitive skills of the animals (at the time of this test young mice were 6 weeks old, old mice were 20–22 months old). Next, the mice received daily intraperitoneal injections of the glycogen phosphorylase inhibitor BAY U6751 (BAY; 25 μg per 1 g tissue, in 0.9% NaCl) or saline (0.9% NaCl). The working concentration of BAY was estimated based on our initial in vitro results (Figure S1). After 14 days, the NOR and NOL test was repeated (young mice were 8 weeks old, old mice were 20–22 months old). To test whether BAY induced stress or affected locomotor skills, the sucrose preference and Rotarod tests were performed following the second round of the NOR. Four animal groups were analyzed: young control (**Y‐CTR**), young treated with BAY (**Y‐BAY**), old control (**O‐CTR**), and old treated with BAY (**O‐BAY**). The schematic representation of the experimental design is depicted in Figures 1a and 2a. The body mass of the animals was monitored throughout the experiment. Animals were euthanized the next day after the last behavioral test.

### 5.3 2‐Novel object recognition test (NOR)

The NOR procedure was performed without the habituation step according to M. Leger et al. (Leger et al., 2013) (see Figure S2). A 33 cm × 33 cm × 20 cm (length, width, height, respectively) box made from white opaque material and with top lightening (5 lux intensity) was used. The test consisted of the familiarization session—in this session each mouse was placed in the box, and allowed to explore two identical objects for 10 min (T1); the intersession interval (ISI) = 6 h—the mouse was returned to its home cage; and the test session—the mouse was placed in the box again, with one familiar and one novel object (T2). The novel object was different in shape, texture and color. To avoid stress resulting from removing only one animal from the cage, each pair of animals from one cage was tested simultaneously in identical boxes, with identical set of objects. After each session, the box and the objects were thoroughly cleaned with ethanol and distilled water to remove any residual scent. The behavior of mice during T1 and T2 was recorded by digital camera located approximately 1.5 m above the box. After 14 days of the pharmacological treatment, the NOR test was repeated with novel objects. For the detailed representation of the NOR test design, please refer to Figure S2.

### 5.4 Novel object location test (NOL)

For the NOL, we used the same behavioral setup and additional animal care procedures between test steps, as described in Methods 5.3. The NOL were performed according to Denninger et al. (2018). At Day 0 and Day 14, the NOL test consisted of the 10‐min habituation to the empty box 24 h before training (T0); the familiarization session—in this session each mouse was placed in the box, and allowed to explore two identical objects (T1, 10 min); the ISI = 20 min; the NOL test session—a mouse was placed in the box again, where one of the familiar objects changed location (T2, 10 min). After 14 days of the pharmacological treatment, the NOL tests were repeated with novel objects. For the detailed representation of the tests design, please refer to Figure 2a.

### 5.5 Behavioral analysis of the NOR test and the NOL test

Video recordings of mice behavior during the NOR and NOL tests were analyzed using a self‐developed analysis system. Exploration time was counted whenever the nose of an animal was at least 2 cm from the object, facing toward the object. When an animal climbed the object with all four pawns, the exploration time was not counted. The analysis continued until the criterion of total 20 s of exploration (sum for both objects) was reached. In case of not reaching 20 s of total exploration criterion within 10 min of the familiarization or test session, the animal was excluded from further analysis. The amount of time the mouse spent exploring a new object and time from placing the mouse in the box to the first exploration have been used to measure, respectively, the hippocampus‐ and cortex‐dependent cognitive skills such as memory and recognition.

### 5.6 Sucrose preference test (SPT)

The SPT test was performed in the dark phase of the 24‐h cycle. To decrease the likelihood of bias resulting from neophobia to sweet taste, mice were given 2.5% sucrose solution for 2 h instead of water 1 day before the actual test. The SPT test was performed before and after 14 days of BAY administration. The mice were given free‐choice access to 1% sucrose solution and water that were provided in identical bottles for 12 h. To eliminate possible bias from side preference, the positions of the bottles were changed after 6 h of the test. The consumption of water and sucrose solution was estimated by weighing the bottles. The percentage of sucrose preference was calculated using the following equation:

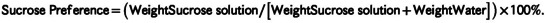




Other conditions of the test were as previously described by Strekalova et al. (2011).

### 5.7 Rotarod test

To measure the motor skills of mice, the widely used Rotarod test was performed. After 1 hour of habituation to the room where the test took place, mice were gently placed on the rubber shaft, which was set to initial rotational motion (10 rpm). The shaft rolling speed was gradually increasing (0.125 rpm/s). The test lasted for maximum 240 s. The amount of time the mouse spent on the spinning shaft was used to determine the animal's motor abilities. The test was performed 4 times for each animal, and the average time spent on the shaft was used for further analysis.

### 5.8 Tissue preparation

For the dendritic spine analysis (Methods 5.9), mice were anesthetized (ketamine/xylazine) and transcardially perfused with 1.5% paraformaldehyde in PBS. The brains were dissected, the hippocampi and frontal cortex separated in an ice‐cold phosphate buffer, and sliced using a vibratome. For the proteomic and LC–MS analyses (Methods 5.11 and 5.14), animals were anesthetized with isoflurane and decapitated. The brains were dissected, the hippocampi and frontal cortex separated, frozen immediately in liquid nitrogen, and kept at −80°C for further analysis. For immunofluorescent staining (Methods 5.12), hippocampi were immersed in alcoholic fixative (1 methanol: 3 ethanol 95%) for 20 min at 4°C and subsequently kept at −20°C. Fixed hippocampi were washed in 99.8% ethanol (4°C) and processed through several mixtures of ethanol and increasing concentration of polyester wax (Science Services) until they were embedded in pure wax. The embedded tissue was cut into 4 μm‐thick sections on rotary microtome (Leica, RM 2255) and mounted on glass slides (Superfrost Plus slides, Menzel Glaser).

### 5.9 DiI staining of brain slices

To visualize changes in the shape of dendritic spines, 1,1′‐dioctadecyl‐3,3,3,3′‐tetramethylindocarbocyanine perchlorate (DiI) staining was performed in slices (140 μm thick) of different brain structures (Bączyńska et al., 2021). The slices were allowed to recover for at least 1.5 h at room temperature. Random dendrite labeling was performed using 1.6 μm tungsten particles (Bio‐Rad) coated with propelled lipophilic fluorescent dye (DiI; Invitrogen) and delivered to the cells by gene gun (Bio‐Rad) bombardment. Images of dendrites in different brain regions were acquired under 561 nm fluorescent illumination using a confocal microscope (63× objective, 1.4 NA) at a pixel resolution of 1024 × 1024 with a 3.43 zoom, resulting in a 0.07 μm pixel size.

### 5.10 Morphometric analysis of dendritic spines

The analysis of the dendritic spine morphology and calculation of changes in spine parameters were performed as described previously (Krzystyniak et al., 2019). The images acquired from the brain slices were processed using ImageJ software (National Institutes of Health, Bethesda, MD, USA) and analyzed semi‐automatically using the custom‐written SpineMagick software (patent no. WO/2013/021001) and 3dSpAn software for three‐dimensional dendritic segment reconstruction (Basu et al., 2016). The spine length was determined by measuring the curvilinear length along a fitted virtual skeleton of the spine. The fitting procedure was performed by looking for a curve along which integrated fluorescence was at a maximum. The head width was defined as the diameter of the largest spine section while excluding the bottom part of the spine (1/3 of the spine length adjacent to the dendrite). Based on that we calculated a scale‐free parameter length‐to‐width ratio, which effectively describes the spine shape (Michaluk et al., 2011). Dendritic segments of at least 4 animals per condition were morphologically analyzed resulting in 1100–1250 spines per group. To determine the spine density, approximately 1000–1500 μm of dendritic length was analyzed per experimental group (9–14 cells per group).

### 5.11 Proteomic analysis

The sample preparation and proteomic measurements were performed as described before (Drulis‐Fajdasz et al., 2021) using two groups of female C57BL/10 J mice: young (8‐week‐old; *n* = 10) and old (20‐ to 22‐month‐old; *n* = 10), which were treated with BAY (*n* = 5/group) or saline (*n* = 5/group), as described in Methods 5.2. The overview of the proteomic experiment is presented in Figure 5a.

In brief, hippocampi were dissected from the mice and the samples were prepared by tissue lysis in SDS containing buffer, followed by a two‐step consecutive protein digestion by multi‐enzyme digestion filter aided sample preparation (MED‐FASP) protocol using Lys‐C and trypsin (Wiśniewski et al., 2020). The obtained peptides were separately analyzed by LC–MS/MS, and MaxQuant (MQ) software (Max Planck Institute, Martinsried, Germany) was used for spectra searching. Specific protein concentrations were calculated by the “Total Protein Approach” (TPA) using raw intensity MQ output (Wiśniewski & Rakus, 2014). Statistical analysis was conducted using Perseus software v.1.6.14.0 (Max Planck Institute for Biochemistry). The cutoff values of 1.2‐fold for up‐and down‐regulated proteins between samples were established. The following comparisons were performed: BAY‐treated old mice (O‐BAY) versus old saline‐treated control animals (O‐CTR), BAY‐treated old mice versus young saline‐treated animals (Y‐CTR), old controls versus young controls, and BAY treated young mice (Y‐BAY) versus young controls, to fully characterize the effect of BAY treatment on the mouse proteome.

### 5.12 Immunofluorescent studies

Before immunolabeling, dewaxed and rehydrated sections were blocked for 1 h with 1% Bovine Serum Albumin (BSA, cat nr: 9048‐46‐8, Merck) and 0.1% Triton X‐100 (Tx, cat nr: 9002‐93‐1, Sigma‐Aldrich) in phosphate‐buffered saline (PBS) pH 7.4. Then, slices were incubated overnight at 4°C with primary antibodies in PBS, 0.1% BSA, 0.001% Tx: anti‐β‐Tubulin Isotype III antibody (1:500, cat nr: 302302, Synaptic Systems); anti‐β‐Tubulin Isotype III antibody (1:500, cat nr: T5076, Sigma‐Aldrich); anti‐GAD67 (Gad1; 1:200, cat nr: MA5‐31377, Invitrogen); anti‐GluR2 (Gria2; 1:200, cat nr: PA5‐19496, Invitrogen); and anti‐CaMKIV (Camk4; 1:200, cat nr: PA1‐542, Invitrogen), respectively. Subsequently, slices were incubated for 2.5 h at RT with secondary antibodies in PBS, 0.1% BSA (1:2000 AlexaFluor 633 goat anti‐mouse cat nr: A21046 and 1:2000 AlexaFluor 488 goat anti‐rabbit, cat nr: A11034, Invitrogen; 1:2000 AlexaFluor 633 goat anti‐rabbit, cat nr: A21071, Invitrogen and 1:2000 AlexaFluor 488 goat anti‐mouse, cat nr: A11001, Invitrogen), respectively. Finally, glass slides were mounted with Fluoroshield with DAPI (cat nr: F6057, Sigma‐Aldrich). Between each step of the immunostaining procedure, sections were washed with PBS (3 × 10 min, RT).

### 5.13 IF image analysis

IF‐stained hippocampal slices isolated from 4 animal groups (Y‐CTR, Y‐BAY, O‐CTR, O‐BAY) were imaged using identical acquisition parameters (set individually for each analyzed protein: Camk4, Gria2, or Gad1) and examined with the Confocal Laser Scanning Microscope FV3000 (Olympus) with 60× objective, at 2048 × 2048 picture resolution. The analysis of relative fluorescence was performed within three regions of hippocampi (SP, *stratum pyramidale*; SR, *stratum radiatum*; *stratum lacunosum‐moleculare*, SLM). The mean fluorescence intensity was calculated using the ImageJ software (Schneider et al., 2012). The average grey value within the selection was counted with threshold in over/under mode (pixel values range 640–4095). To exclude tissue autofluorescence background that usually arise in tissue samples obtained from old animals, the pixel values were corrected by subtracting the average background fluorescence.

### 5.14 LC–MS analysis

The quantification of BAY in the hippocampus and cortex was performed with LC–MS analysis. The sample preparation and experimental instrument parameters were described previously (Pudelko‐Malik et al., 2022). Briefly, frozen samples of the hippocampus and cortex tissue were weighted to equal sample size 30 mg and extracted with ice‐cold methanol. Homogenization was carried out using the TissueLyser LT (QIAGEN) at 25 Hz for 5 min. Then, the samples were centrifuged at 10,000 rcf (15 min, 4°C). The supernatants were evaporated at 1400 rcf (2 h, 45°C), and the dried samples were then dissolved in 100 μL of methanol and mixed for 3 min. Then, they were centrifuged at 14,000 rcf (5 min, 4°C), and 50 μL of each supernatant was transferred into the glass insert and immediately analyzed by LC–MS. Each sample was proceed with internal standard (IS) BAY R3401 (Sigma Aldrich) in concentration of 25 ng/mL. The mass spectrometer Synapt G2 Si Q‐TOF MS with an electrospray ion source (ESI) was combined with the Acquity UPLC I‐class chromatographic system (Waters). Chromatographic separation was performed on the Acquity UPLC CSH C18 (2 × 100 mm, 1.7 μm, Waters) analytical column. The mobile phase comprised of water (A) and methanol (B) with the addition of 0.1% formic acid. The MassLynx software (version 1.60.1774, Waters) with the QuanLynx application were used for data acquisition and quantitative analysis, respectively. The limit of detection (LOD) and limit of quantification (LOQ) were calculated using the calibration curve and were 7.76 and 23.29 ng/mL, respectively. In the presented analytical method, precision was expressed as the coefficient of variation (CV). In order to meet the acceptance criteria of the European Medicines Agency (EMA) Guidelines, the CV did not exceed ±20% for lower limit of quantification (LLOQ) and ±15% for higher value of concentration.

### 5.15 Statistical analysis

All tests for 4 groups of animals were analyzed: Y‐CTR, Y‐BAY, O‐CTR, and O‐BAY. In behavioral tests; *n* = 6–9 animals for each group were analyzed. In the immunolabeling and Dil staining, *n* = 4 animals for each group, and the proteomic data we determined for *n* = 6 animals for each group. For statistical analysis, we used the parametric unpaired Student's *t*‐test and/or the Kolmogorov–Smirnov test (*D* ‐ max deviation; ks ‐ Kolmogorov–Smirnov test statistic; *p*—probability). The statistical significance of differences was indicated by asterisks as follows: *p < 0.05, **p < 0.01, ***p < 0.001. For the LC–MS measurements, precision was calculated by coefficient of variation (CV).

### 5.16 Graphics

All figures were prepared with BioRender.com.

## AUTHOR CONTRIBUTIONS

Conceptualization: Dominika Drulis‐Fajdasz and Dariusz Rakus; Methodology: Dominika Drulis‐Fajdasz, Adam Krzystyniak, Agata Pytyś, Natalia Pudełko‐Malik, Tomasz Wójtowicz, Jerzy Ł. Wiśniewski, Jacek R. Wiśniewski; Investigation: Dominika Drulis‐Fajdasz, Adam Krzystyniak, Alicja Puścian, Agata Pytyś, Kinga Gostomska‐Pampuch, Natalia Pudełko‐Malik, Jerzy Ł. Wiśniewski, Arkadiusz Miążek, Tomasz Wójtowicz, Kamila Duś‐Szachniewicz; Visualization: Dominika Drulis‐Fajdasz, Adam Krzystyniak, Natalia Pudłeko ‐Malik, Tomasz Wójtowicz, Kamila Duś‐Szachniewicz; Supervision: Piotr Młynarz, Jakub Włodarczyk, Jacek R. Wiśniewski, Dariusz Rakus; Writing—original draft: Dominika Drulis‐Fajdasz, Adam Krzystyniak, Natalia‐Pudełko‐Malik, Kamila Duś‐Szachniewicz, Agnieszka Gizak, Dariusz Rakus; Writing—review & editing: Dominika Drulis‐Fajdasz, Piotr Młynarz, Arkadiusz Miążek, Alicja Puścian, Tomasz Wójtowicz, Agnieszka Gizak, Jacek R. Wiśniewski, Dariusz Rakus.

## CONFLICT OF INTEREST STATEMENT

The authors declare no conflict of interest.

## Supporting information


Figure S1.
Figure S2.
Figure S3.
Figure S4.
Figure S5.
Figure S6.

Table S1.

Table S2.

## Data Availability

The data that support the findings of this study are openly available in Proteome Xchange Consortium via the PRIDE partner repository with the dataset identifier PXD025978, file UU6‐UU15. The authors declare that the data supporting the findings of this study are available within the paper and its supplementary information files.
