# Correction to “Glycogen phosphorylase inhibition improves cognitive function of aged mice”

**DOI:** 10.1111/acel.14026

**Published:** 2023-10-27

Drulis‐Fajdasz, D., Krzystyniak, A., Puścian, A., Pytyś, A., Gostomska‐Pampuch, K., Pudełko‐Malik, N., Wiśniewski, J., Młynarz, P., Miazek, A., Wójtowicz, T., Włodarczyk, J., Duś‐Szachniewicz, K., Gizak, A., Wiśniewski, J., Rakus, D., (2023). Glycogen phosphorylase inhibition improves cognitive function of aged mice. Aging Cell, 22(9), e13928.

Krzystyniak Adam (second co‐author) affiliation is incorrect. The previous list of authors with affiliations was:

“Dominika Drulis‐Fajdasz ^1^, Adam Krzystyniak ^2^, Alicja Puścian ^3^, Agata Pytyś ^2^, Kinga Gostomska‐Pampuch ^4 5^, Natalia Pudełko‐Malik ^6^, Jerzy Ł Wiśniewski ^6^, Piotr Młynarz ^6^, Arkadiusz Miazek ^7^, Tomasz Wójtowicz ^2^, Jakub Włodarczyk ^2^, Kamila Duś‐Szachniewicz ^8^, Agnieszka Gizak ^1^, Jacek R Wiśniewski ^5^, Dariusz Rakus ^1^

Affiliations.

1 Department of Molecular Physiology and Neurobiology, University of Wroclaw, Wroclaw, Poland.

2 Laboratory of Cell Biophysics, Nencki Institute of Experimental Biology, Polish Academy of Sciences, Warsaw, Poland.

3 Nencki‐EMBL Partnership for Neural Plasticity and Brain Disorders – BRAINCITY, Nencki Institute of Experimental Biology, Polish Academy of Sciences, Warsaw, Poland.

4 Department of Biochemistry and Immunochemistry, Wroclaw Medical University, Wroclaw, Poland.

5 Biochemical Proteomics Group, Department of Proteomics and Signal Transduction, Max Planck Institute of Biochemistry, Martinsried, Germany.

6 Department of Biochemistry, Molecular Biology and Biotechnology, Faculty of Chemistry, Wroclaw University of Science and Technology, Wroclaw, Poland.

7 Laboratory of Tumor Immunology, Hirszfeld Institute of Immunology and Experimental Therapy, Polish Academy of Sciences, Wroclaw, Poland.

8 Department of Clinical and Experimental Pathology, Institute of General and Experimental Pathology, Wroclaw Medical University, Wroclaw, Poland.”

This should have read:

“Dominika Drulis‐Fajdasz ^1^, Adam Krzystyniak ^2^, Alicja Puścian ^3^, Agata Pytyś ^4^, Kinga Gostomska‐Pampuch ^5 6^, Natalia Pudełko‐Malik ^7^, Jerzy Ł Wiśniewski ^7^, Piotr Młynarz ^7^, Arkadiusz Miazek ^8^, Tomasz Wójtowicz ^4^, Jakub Włodarczyk ^4^, Kamila Duś‐Szachniewicz ^9^, Agnieszka Gizak ^1^, Jacek R Wiśniewski ^6^, Dariusz Rakus ^1^

Affiliations.

1 Department of Molecular Physiology and Neurobiology, University of Wroclaw, Wroclaw, Poland.

2 Laboratory of Molecular Bases of Aging, Nencki Institute of Experimental Biology, Polish Academy of Sciences, Warsaw, Poland.

3 Nencki‐EMBL Partnership for Neural Plasticity and Brain Disorders – BRAINCITY, Nencki Institute of Experimental Biology, Polish Academy of Sciences, Warsaw, Poland.

4 Laboratory of Cell Biophysics, Nencki Institute of Experimental Biology, Polish Academy of Sciences, Warsaw, Poland.

5 Department of Biochemistry and Immunochemistry, Wroclaw Medical University, Wroclaw, Poland.

6 Biochemical Proteomics Group, Department of Proteomics and Signal Transduction, Max Planck Institute of Biochemistry, Martinsried, Germany.

7 Department of Biochemistry, Molecular Biology and Biotechnology, Faculty of Chemistry, Wroclaw University of Science and Technology, Wroclaw, Poland.

8 Laboratory of Tumor Immunology, Hirszfeld Institute of Immunology and Experimental Therapy, Polish Academy of Sciences, Wroclaw, Poland.

9 Department of Clinical and Experimental Pathology, Institute of General and Experimental Pathology, Wroclaw Medical University, Wroclaw, Poland.”

We apologize for this error.

