# Supplementary material for: Glycogen phosphorylase inhibition improves cognitive function of aged mice
Source: Aging Cell. 2023 Jul 31;22(9):e13928. doi: 10.1111/acel.13928 (PMC10497847; doi:10.1111/acel.13928)
Supplement: Supplementary file 1 — Figure S1. Figure S2. Figure S3. Figure S4. Figure S5. Figure S6. [file ACEL-22-e13928-s003.docx]

**Supplementary Materials**

**Supplementary Figure 1**

**Dose-response effect of BAY on the LTP formation.**

In the initial stage of this project, we studied changes in the long-term synaptic plasticity depending on the dose of bath-applied BAY 6751 (BAY), in acute brain slices of young (2-3-month-old) Wistar rats. To this end, we performed electrophysiological recordings of the evoked field excitatory postsynaptic potentials (fEPSPs) in the hippocampal CA1 region, to characterize the excitatory synaptic transmission. BAY was bath-applied throughout the experiment. We found that the magnitude of LTP measured 90 minutes after the induction was significantly lowered by BAY in a dose-dependent manner (**Supplementary Fig. 1 A-F).** Specifically, concentrations of 5-50 µM, but not 3 µM, significantly affected the LTP magnitude (One-Way ANOVA with multiple comparisons, F_(4, 22)_=12.11; p<0.001; n=3-7 slices per group, **Supplementary Fig. 1 F**).

We compared the amplitudes of the fEPSPs in response to a wide range of stimuli (0-300 µA), applied to Schaffer collaterals 15 minutes before and again 90 minutes post the LTP induction **(Supplementary Fig. 1 G-K**). As expected, in the control conditions, the fEPSP amplitudes were significantly larger after the LTP induction (Two-Way ANOVA, F_(15, 180)_=4.690; p<0.001; n=7 slices per group, **Supplementary Fig. 1 G**). Slices treated with 3 µM BAY throughout the experiment exhibited no changes in the fEPSP curve observed after LTP (Two-Way ANOVA, F_(15, 89)_=1.342 p=0.19; n=4 slices). In contrast, in all slices treated with 5-50 µM BAY, the synaptic responses were significantly reduced 90 minutes post LTP (Two-Way ANOVA results were F_(15, 120)_=12.81; p<0.001; F_(15, 90)_=5.541; p<0.001 and F_(15, 90)_=5.160; p<0.001 respectively).

**Taken together, the bath-applied BAY at doses of 5-50 µM had clearly negative effect on the LTP magnitude in hippocampal slices from young animals.**

**
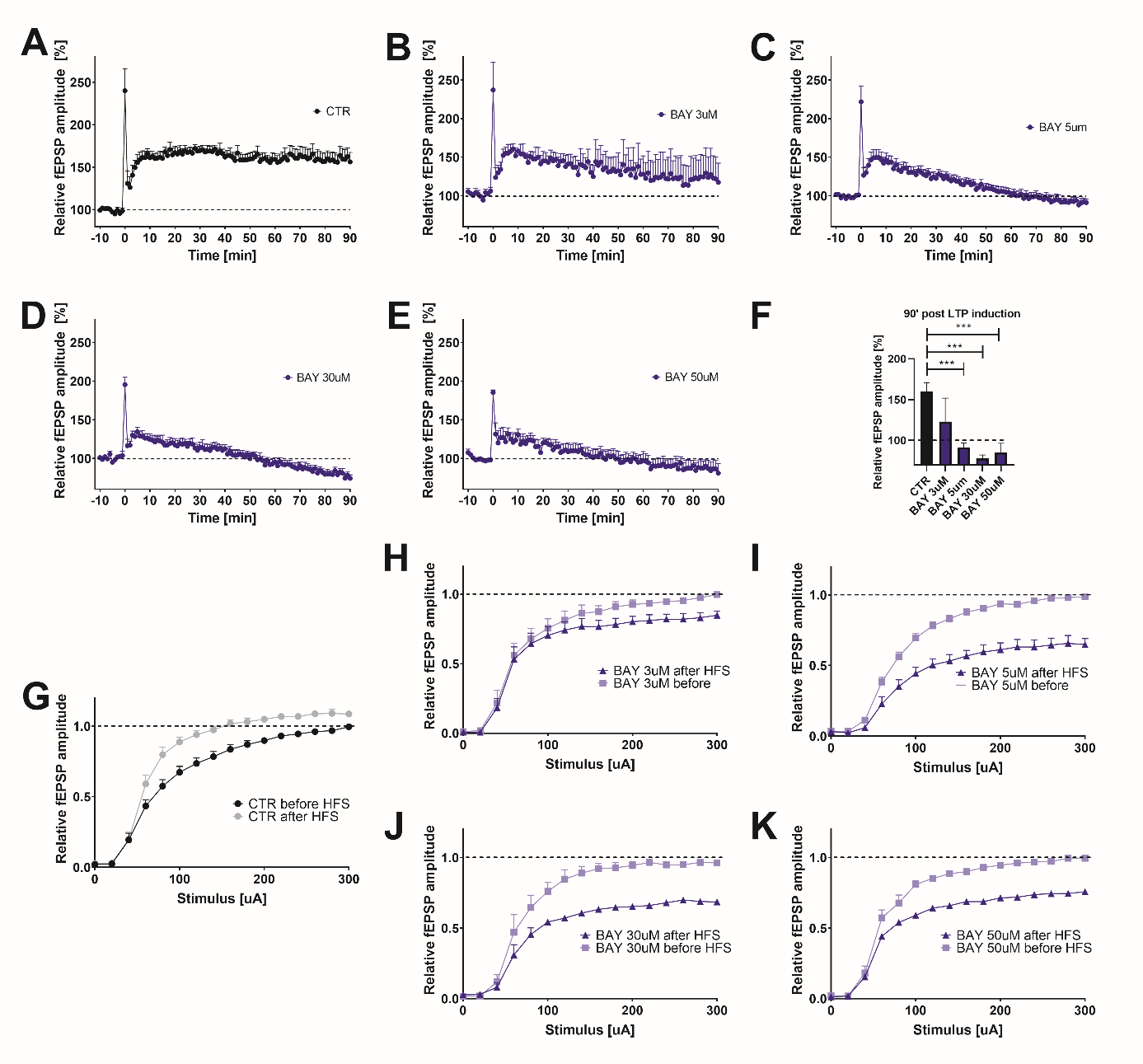
**

**Supplementary Figure 1. Dose-response effect of BAY on the LTP formation. A-F)** Average time course of fEPSP amplitudes following high-frequency stimulation protocol (HFS; 4x100Hz) applied at time 0 in the control slices **(A)** and in the slices treated with different concentrations of bath-applied BAY **(B-E**). **F)** Average fEPSP amplitudes recorded 90 minutes post HFS in the same experiments as shown in A-E.

Average fEPSP amplitudes recorded in response to a wide range of stimuli applied before and 90 minutes after the HFS in the control slices **(G)** and in the slices treated with different concentrations of bath-applied BAY **(H-K)**.

**Method**

**Electrophysiological recordings**

All the experimental procedures were approved by the Local Ethics Committee. The animals were anesthetized with isoﬂurane and decapitated. The hippocampi were dissected and cut into 350 µm thick slices using a vibratome (5100 mz, Campden Instruments, USA) in an ice-cold buffer that contained: 92 mM N-methyl-D-glucamine, 2.5 mM KCl, 1.25 mM NaH_2_PO_4_, 30 mM NaHCO_3_, 20 mM HEPES, 25 mM glucose, 2 mM thiourea, 5 mM Na-ascorbate, 3 mM Na-pyruvate, 0.5 mM CaCl_2_·4H_2_O, and 10 mM MgSO_4_·7H_2_O. pH was set to 7.3–7.4 with concentrated hydrochloric acid as previously described (Ting et al., 2014). Slices were subsequently allowed to recover in an artificial cerebrospinal fluid (“holding solution”, 32°C, 15 min) containing: 92 mM NaCl, 2.5 mM KCl, 1.25 mM NaH_2_PO_4_, 30 mM NaHCO_3_, 20 mM HEPES, 25 mM glucose, 2 mM thiourea, 5 mM Na-ascorbate, 3 mM Na-pyruvate, 2 mM CaCl_2_·4H_2_O, and 2 mM MgSO_4_·7H_2_O, pH 7.4. Slices were then stored until the end of the experiments in an aCSF that contained: 125 mM NaCl, 25 mM NaHCO3, 2.6 mM KCl, 1.25 mM NaH_2_PO_4_, 2.0 mM CaCl_2_, and 20 mM glucose, pH 7.4. All the solutions were oxygenated with carbogen (95 % O_2_, 5 % CO_2_). Recordings were made in the aCSF alone (control samples) or in aCSF supplemented with 3, 5, 30 or 50 µM BAY U6751.

Schaeffer collateral axons were stimulated with a concentric bipolar electrode (ME20688, Microprobes, USA; A385 stimulus isolator, WPI, USA; basal stimulation 0.1 Hz, 0.3 ms). Next, LTP was induced using the high frequency stimulation protocol (100 Hz for 1 s, repeated 4 times every 10 s). AMPAR-mediated fEPSPs were recorded with glass micropipettes filled with the aCSF (1-3 MΩ resistance) in the stratum radiatum of the CA1 region (150 – 200 μm from the stratum pyramidale). All the recorded signals were amplified (Axopatch 200B amplifier, Molecular Devices, USA), low-pass-filtered at 10 kHz and sampled at 20 kHz (Digidata 1322, Molecular Devices, USA). The data analysis was performed using the pClamp10.7.0.3 software (Molecular Devices, USA) and AxoGraphX software (developed by John Clements).

Ting, J. T., Daigle, T. L., Chen, Q., & Feng, G. (2014). Acute brain slice methods for adult and aging animals: application of targeted patch clamp analysis and optogenetics. Methods in Molecular Biology (Clifton, N.J.), 1183, 221–242.

**Supplementary Figure 2**

**
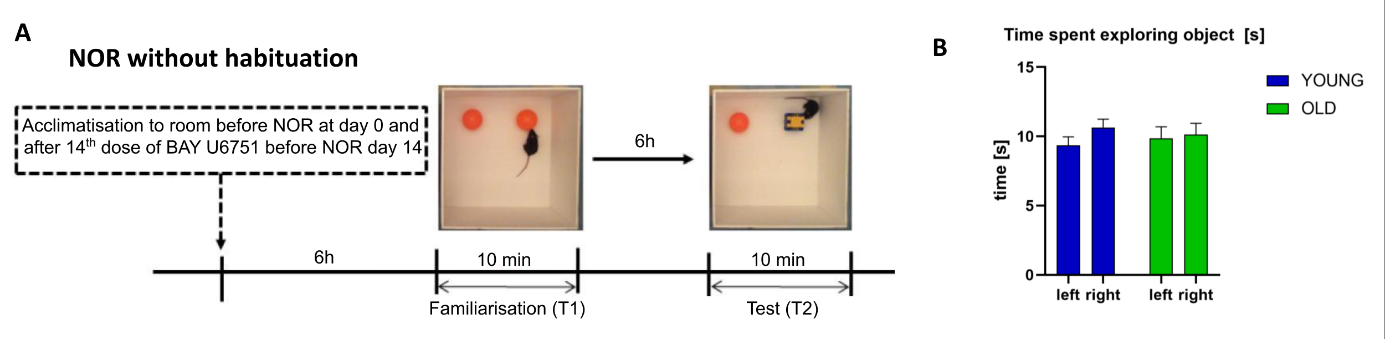
**

**Supplementary Figure 2**. **A**) **Time schedule of an individual 2-Novel Object Recognition test (NOR) performed at day 0 and day 14**. The familiarization session (T1) was performed 6 h after the last handling (day 0) and the last dose of glycogen phosphorylase inhibitor BAY U6751. Animals explored the open box with two identical objects for 10 min. The test session (T2) with a novel object exposition was performed after 6h break. **B**) Average time spent on exploration of left or right objects during T1 session indicated the appropriateness of the NOR test: neither young nor old animals revealed object site preference in the NOR test at day 0 and day 14.

**Supplementary Figure 3**


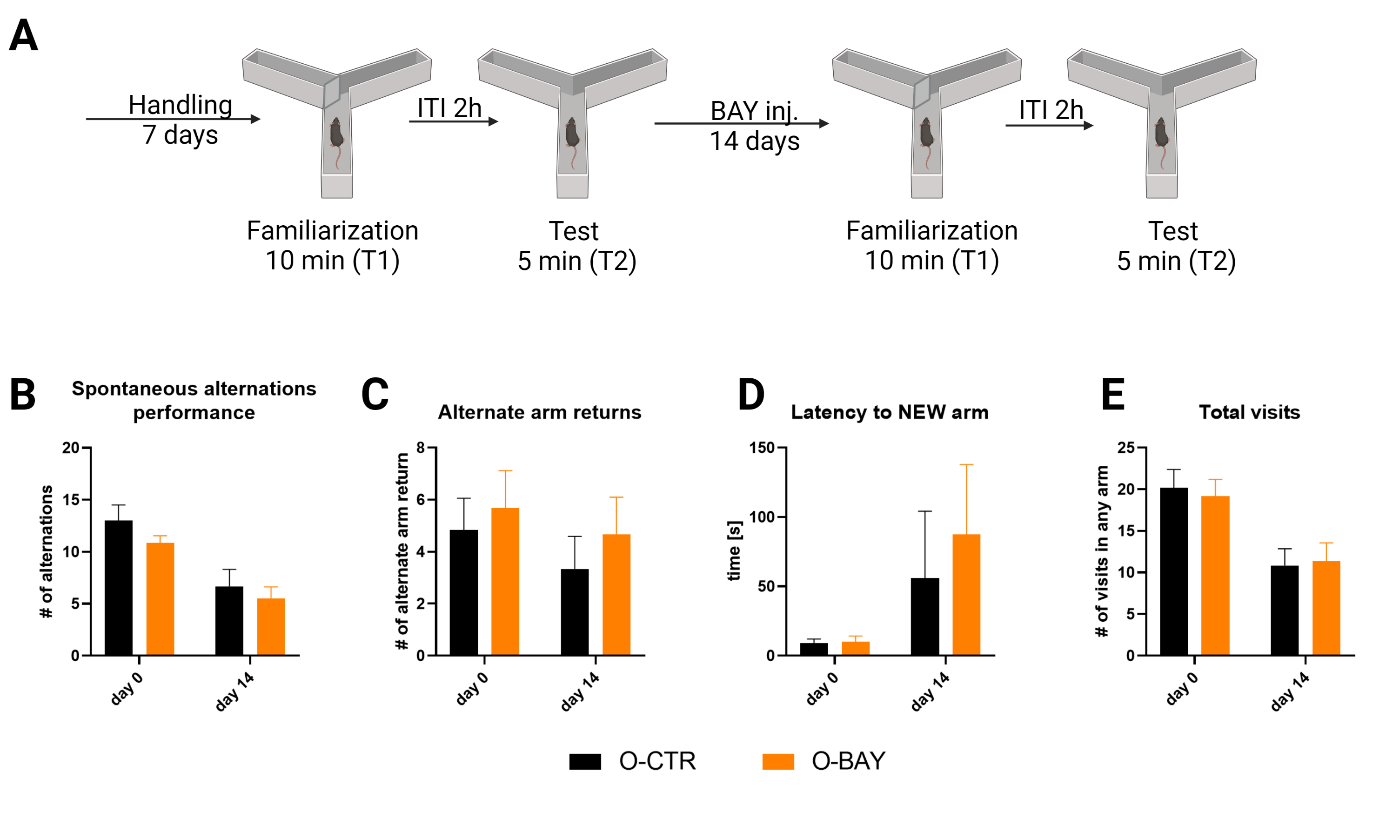


**Supplementary Figure 3. Spatial memory testing in Y-maze.** Inhibition of glycogen phosphorylase did not improve working memory in the Y-maze test, in 20-22-month-old mice. Schematic representation of the experiment **(A)**. Number of spontaneous alterations **(B)** and alternate arm returns **(C)** during the test session (T2) performed before (day 0) and after (day 14) the BAY or saline daily injections. Latency to the first entry into a new arm **(D)**, and the ratio of the latency to the first exploration between day 14 and day 0 **(E)**. We observed that the BAY treated mice did not respond with increased spontaneous alternation performance and alternate arm returns compared to the vehicle treated mice. The number of subjects in each experimental group n=6. The statistically significant changes between groups are indicated (*p < 0.05; **p < 0.01).

**Method: memory testing in Y-maze**

The study employed the Y-maze paradigm to investigate spatial memory in aged (20-22-month-old) C57BL/6J mice. The maze consisted of three enclosed light grey Plexiglas arms, each measuring 21 cm in length, 15.5 cm in width, and 7 cm in height, with extra maze visual cues. The testing protocol consisted of two trials, separated by a 2-hour intertrial interval (ITI). Mice were transported to the behavioral testing room in their home cages, and subjected to a familiarization trial during which they were allowed to explore the maze for 5 minutes, with one arm closed (novel arm). In the second trial (test trial), mice could explore all three arms of the maze freely. The time spent in each arm was recorded and analyzed using a computer tracking system (ToxTrac). The time spent in the novel arm was expressed as a percentage of the total time spent in all three arms during the retrieval trial. After 14 days, following intraperitoneal injections of either BAY-U6751 or saline (vehicle), the mice were tested again. After the Y-maze testing, the mice were euthanized by cervical dislocation, and their brains were dissected for electrophysiological analysis.

**Supplementary Figure 4**

**LTP in hippocampal slices isolated from old mice after the Y-maze test**

6 hours after the Y-maze electrophysiological recordings of the evoked field excitatory postsynaptic potentials (fEPSPs) were performed in acute hippocampal brain slices of CA1 region, to describe the excitatory synaptic transmission.

The amplitudes of the fiber volley values recorded in response to a wide range of stimuli (0-300 µA), applied to Schaffer collaterals were significantly smaller in the BAY group compared to the CTR (Two-Way ANOVA, F_(12, 276)_=2.534, p=0.0035; N=6 animals per group, n=12-13 slices per group, **Supplementary** **Fig. 4B**). At the same time, the amplitudes of the AMPAR-mediated fEPSPs were similar in both groups (Two-Way ANOVA, F_(12, 276)_=0.32, p=0.98; **Supplementary Fig. 4A**). Plotting the average fiber volley values versus fEPSP amplitudes showed that the efficacy of synaptic transmission in response to the stimulation of a similar number of presynaptic afferents could be enhanced in the BAY group (**Supplementary Fig. 4C**). Subsequently, the three-dimensional Monte Carlo simulations were performed which incorporated fiber volley, fEPSP, and stimulus intensity. These simulations showed that the amplitude of the AMPAR-mediated fEPSPs normalized to the fiber volley amplitude was not significantly enhanced in the BAY-treated group, compared to controls (Mann-Whitney statistic=94.0, p=0.39; trueDist=0.83, p=0.1586). Thus, stimulation of the same number of presynaptic afferents yielded similar excitatory AMPAR-mediated synaptic drive in the BAY-treated group after the Y-maze learning paradigm.

Next, LTP was recorded after the high frequency stimulation protocol (HFS; 4x 100Hz). 90 minutes after the HFS, the LTP magnitude was significantly larger in the control vs BAY-treated group (unpaired t-test, p=0.001, **Supplementary Fig. 4D-F**) after the Y-maze test.

The fEPSP amplitudes recorded in response to a wide range of stimuli (0-300 µA) 90 minutes after the HFS, were significantly upregulated both in the control and in the BAY-treated group, after the Y-maze test (Two-Way ANOVA, F_(12, 288)_=18.57; p<0.001 and F_(12, 264)_=1.913, p=0.033, respectively; **Supplementary Fig. 4F)**. However, the magnitude of the relative fEPSP amplitude change was significantly less pronounced in the BAY group, compared to the control (Two-Way ANOVA, F_(12, 264)_=7.563; p<0.001; **Supplementary Fig. 4F**).

**
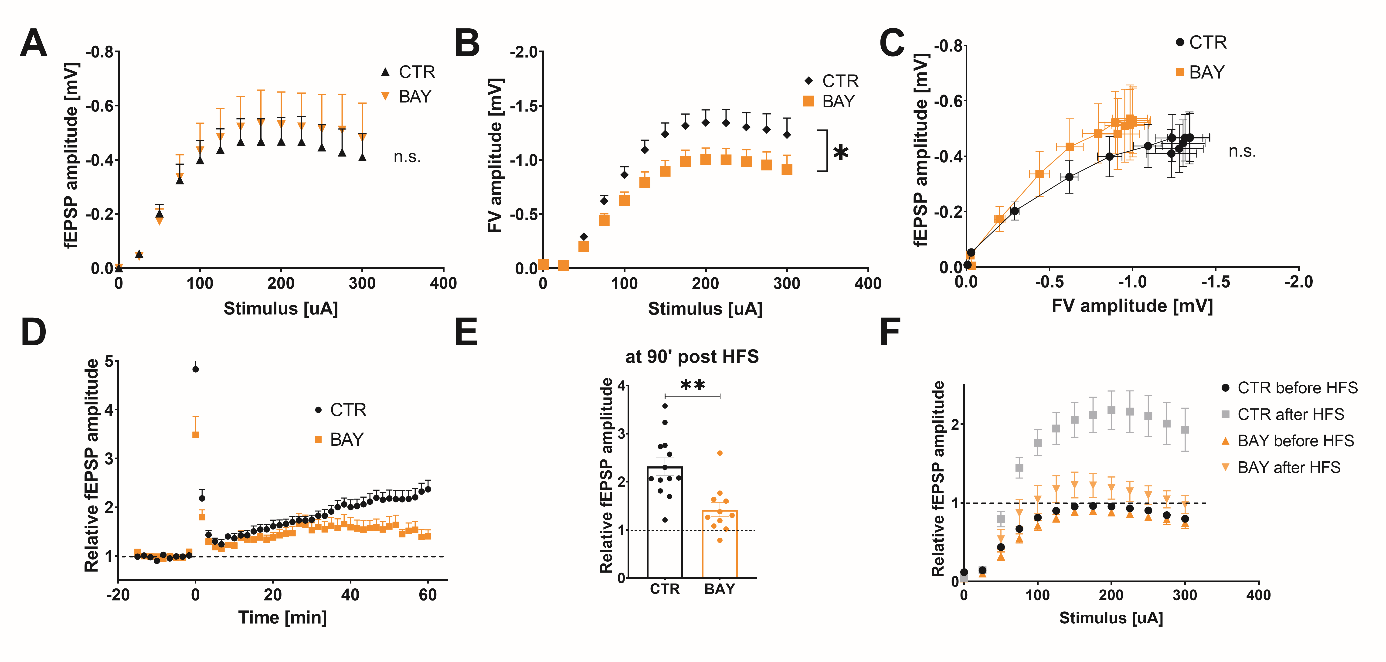
**

**Supplementary Fig. LTP in hippocampal slices isolated from old mice after the Y-maze test.**

Average fEPSP amplitudes **(A)** and fiber volley amplitudes **(B)** recorded in the CA1 region in response to stimulation of Shaffer collaterals, in acute brain slices obtained 6 h post Y-maze training. **C)** Average fEPSP amplitudes plotted versus fiber volley amplitudes recorded in the same hippocampal slices. **D)** Average time course of fEPSP amplitudes following high frequency stimulation protocol (4x 100Hz) applied at time 0. **E)** average fEPSP amplitudes recorded 90 minutes after the HFS. **F)** Average fEPSP amplitudes recorded in response to a wide range of stimuli (0-300 µA) before and 90 minutes after the HFS.

Taken together, a picture emerges suggesting that the glycogen phosphorylase-induced memory improvement determined in the Y-maze test, is reflected by the enhanced hippocampal basal synaptic response and is inversely correlated with the magnitude of the electrically induced LTP.

**This is in accordance with previous reports indicating that the learning-induced synaptic potentiation occluded the HFS-induced LTP** (Li et al., 2005, Whitlock et al., 2006).

**Method: Electrophysiological recordings**

All the experimental procedures were approved by the Local Ethics Committee. The animals were anesthetized with isoﬂurane and decapitated. The hippocampi were dissected and cut into 350 µm thick slices using a vibratome (5100mz, Campden Instruments, USA) in an ice-cold buffer that contained: 92 mM N-methyl-D-glucamine, 2.5 mM KCl, 1.25 mM NaH_2_PO_4_, 30 mM NaHCO_3_, 20 mM HEPES, 25 mM glucose, 2 mM thiourea, 5 mM Na-ascorbate, 3 mM Na-pyruvate, 0.5 mM CaCl_2_·4H_2_O, and 10 mM MgSO_4_·7H_2_O. pH was set to 7.3-7.4 with concentrated hydrochloric acid as previously described (Ting et al., 2014). Slices were subsequently allowed to recover in an artificial cerebrospinal fluid (“holding solution”, 32°C, 15 min) containing: 92 mM NaCl, 2.5 mM KCl, 1.25 mM NaH_2_PO_4_, 30 mM NaHCO_3_, 20 mM HEPES, 25 mM glucose, 2 mM thiourea, 5 mM Na-ascorbate, 3 mM Na-pyruvate, 2 mM CaCl_2_·4H_2_O, and 2 mM MgSO_4_·7H_2_O, pH 7.4. Slices were then stored until the end of the experiments in an aCSF that contained: 125 mM NaCl, 25 mM NaHCO_3_, 2.6 mM KCl, 1.25 mM NaH_2_PO_4_, 2.0 mM CaCl_2_, and 20 mM glucose, pH 7.4. All the solutions were oxygenated with carbogen (95 % O_2_, 5% CO_2_). Recordings were made in the aCSF after 2 hours of the slice recovery. Schaffer collateral axons were stimulated with a concentric bipolar electrode (ME20688, Microprobes, USA; A385 stimulus isolator, WPI, USA; 0.1 Hz, 0.3 ms). Next LTP was induced using high frequency stimulation protocol (100 Hz 1 s long, repeated 4 times every 10 s). AMPAR-mediated fEPSPs were recorded with glass micropipettes filled with the aCSF (1-3 M Ω resistance) in the stratum radiatum of the CA1 region (150 – 200 μm from the stratum pyramidale). All the recorded signals were amplified (Axopatch 200B amplifier, Molecular Devices, USA), low-pass-filtered at 10 kHz and sampled at 20 kHz (Digidata 1344, Molecular Devices, USA). The data analysis was performed using the pClamp10.7.0.3 software (Molecular Devices, USA) and AxoGraphX software (developed by John Clements).

**Monte Carlo simulations**

The statistical significance of the differences between the stimulation current, fiber volley amplitudes and fEPSPs, $V(I)$ , obtained from the electrophysiological experiments, was determined by the Monte Carlo methods. The p values were calculated using a randomization approach (Ruxton et al., 2013). In the first step, we quantified the differences between the measured $V^{A}(I_{i})$ and $V^{B}(I_{i})$ curves using the L­_2_ norm, defined as:

$$L_{2}=\sqrt{{\sum_{i=0}^{N} (V^{A}(I_{i})-V^{B}{(I}_{i}))^{2}}_{,}}$$

where $I_{i}$is the stimulation current for the i-th measurement point and N is the total number of the measurement points. To compute the p values, we created the null-hypothesis ensemble, using the subject randomization. The p value for the difference between two $V\left( I \right)$ curves was calculated as:

$$p_{value}=\frac{\sum_{j=1}^{B} F(L_{2} j\geq L_{2} true)}{B+1}$$

where *F* is the indicator function that takes the value one when its argument is true and zero when it is false, $L_{2} j$ is the $L_{2}$ norm for the -jth element of the null-hypothesis ensemble, $L_{2} true$is the actual value of $L_{2}$ for the analyzed $V(I)$ curves, and B is the number of randomizations (we used B=1000).

Li, Z., Zhou, Q., Li, L., Mao, R., Wang, M., Peng, W., Dong, Z., Xu, L., & Cao, J. (2005). Effects of unconditioned and conditioned aversive stimuli in an intense fear conditioning paradigm on synaptic plasticity in the hippocampal CA1 area in vivo. Hippocampus, 15(6), 815–824.

Ruxton, G. D., & Neuhäuser, M. (2013). Improving the reporting of P-values generated by randomization methods. Methods in Ecology and Evolution, 4(11), 1033–1036.

Ting, J. T., Daigle, T. L., Chen, Q., & Feng, G. (2014). Acute brain slice methods for adult and aging animals: application of targeted patch clamp analysis and optogenetics. Methods in Molecular Biology (Clifton, N.J.), 1183, 221–242.

Whitlock, J. R., Heynen, A. J., Shuler, M. G., & Bear, M. F. (2006). Learning induces long-term potentiation in the hippocampus. Science (New York, N.Y.), 313(5790), 1093–1097.

**Supplementary Figure 5**

**BAY in the brain structures**


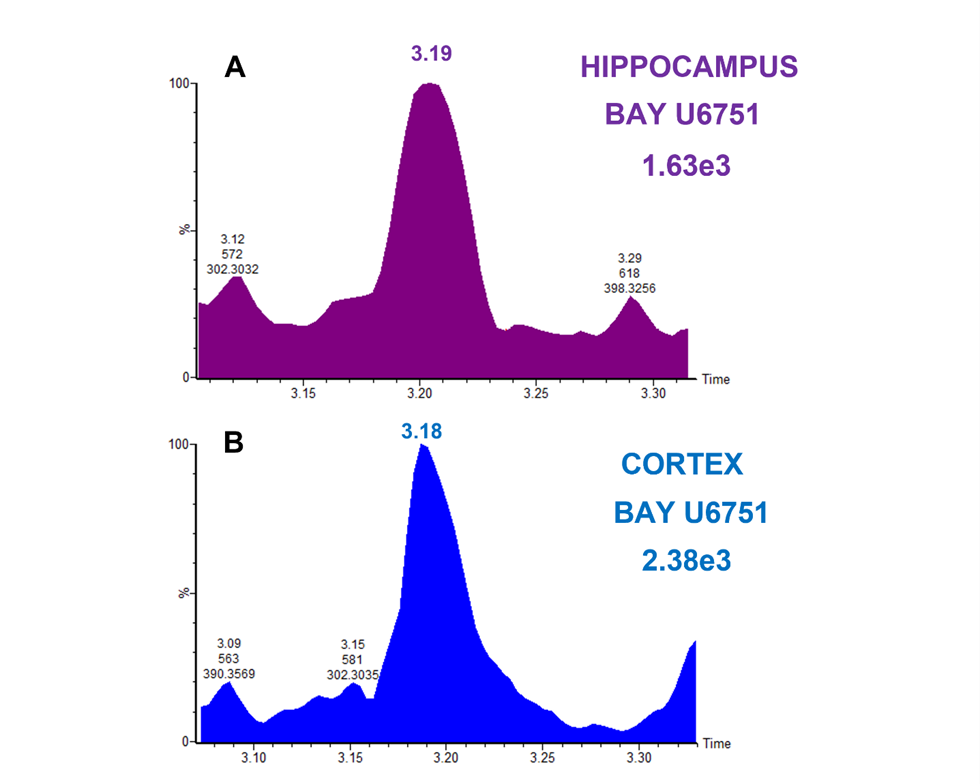


**Supplementary Figure 5.** The representative ion extract chromatograms of the LC-MS analysis of the BAY analyte in the hippocampal (**A**) and cortical tissue extracts (**B**).

**Supplementary Figure 6**

**Glycogen and lactate concentrations in BAY-treated and untreated old mice**

Previous studies on cerebellar and neocortical astrocytes have shown that 1,4-dideoxy-1,4-imino-D-arabinitol (DAB), characterized as inhibitor of glycogen phosphorylase (Pyg), had also an inhibitory effect on glycogen synthase and the prolonged exposure to the inhibitor may reduce glycogen level (Walls et al., 2008). BAY is supposed to have a similar secondary effect (Latsis et al., 2002). Thus, only moderate changes in glycogen and glycogen-derived lactate after the long-term treatment with BAY could be expected**.**

Latsis, T., Andersen, B., & Agius, L. (2002). Diverse effects of two allosteric inhibitors on the phosphorylation state of glycogen phosphorylase in hepatocytes. The Biochemical Journal, 368(Pt 1), 309–316.

Walls, A. B., Sickmann, H. M., Brown, A., Bouman, S. D., Ransom, B., Schousboe, A., & Waagepetersen, H. S. (2008). Characterization of 1,4-dideoxy-1,4-imino-d-arabinitol (DAB) as an inhibitor of brain glycogen shunt activity. Journal of Neurochemistry, 105(4), 1462–1470.


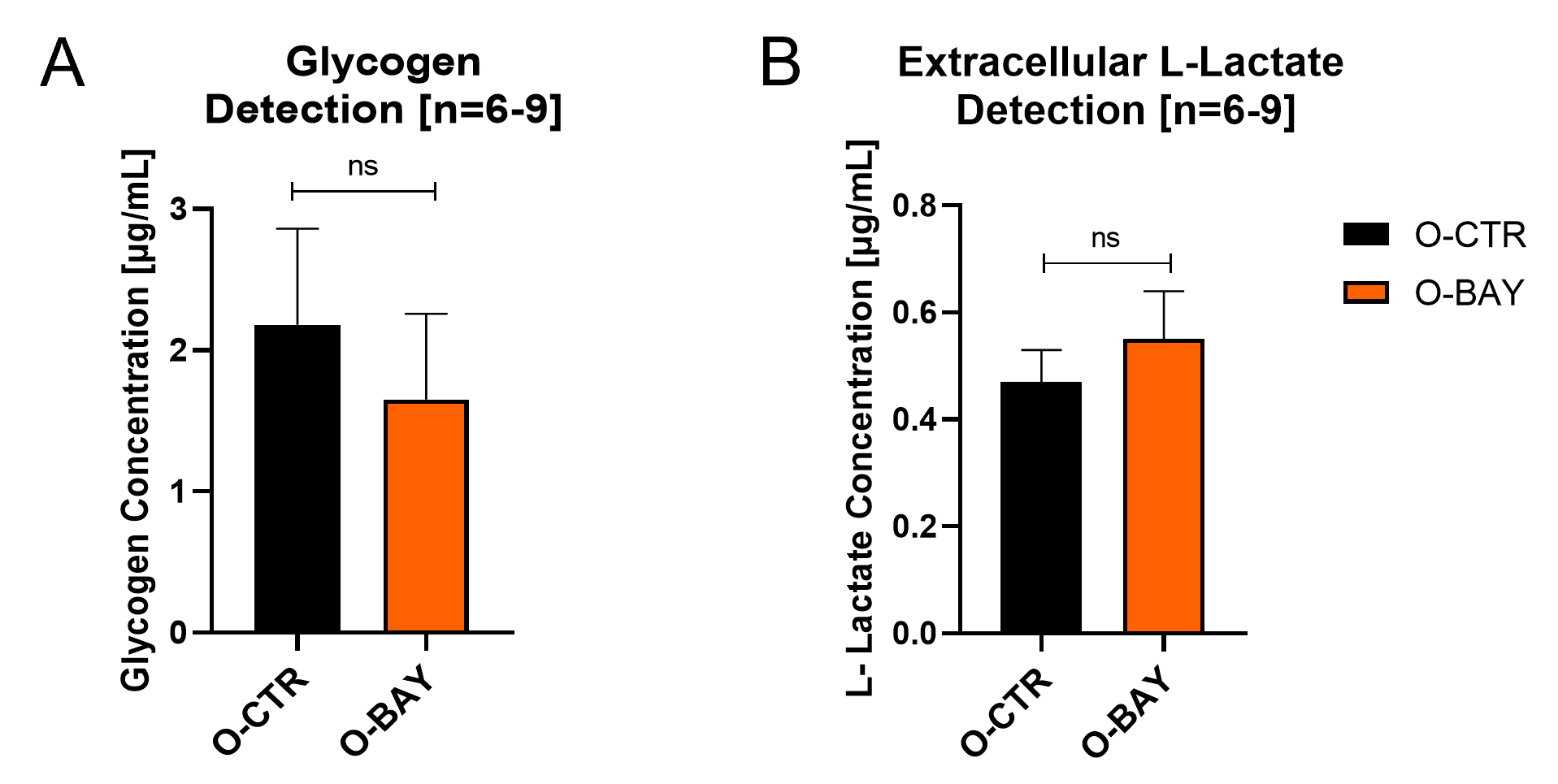


**Supplementary Figure 6. Concentrations of the intracellular lactate (A), and glycogen (B) in the hippocampal tissue.**

Glycogen concentrations [µg/mL] **(A)** and extracellular L-Lactate concentrations [µg/mL] **(B**) were calculated based on 15 biological replicates; control groups (O-CTR; n=6) and experimental groups (O-BAY; n=9), ns – not statistically significant (p-value > 0.05).

**Methods:**

**Determination of Glycogen Concentrations:** The measurement of glycogen levels in the hippocampal tissue was performed using the Glycogen Detection Assay (Promega, Corporation). The experiment was carried out using two independent groups: old animals (O-CTR, n=6), and old animals treated with BAY (O-BAY, n=9). Briefly, 21 mg of frozen hippocampal tissue was homogenized for 30 s in the homogenization buffer (50 mM Tris, pH 7.5 set with 0.6 M HCl), at 8:1 ratio. After the homogenization, 0.125 µL of 600 mM Tris, pH 8.5 was added and the sample was centrifuged 10 000 x g for 10 minutes. Supernatant was collected and incubated for 1 h with glucoamylase. Then, the Glucose Detection Reagent was added and the sample was incubated for 1 h at room temperature. Then the sample luminescence was recorded (Lumi, microdigital). The luminescence intensity was proportional to the glycogen concentration in the samples.

**Determination of the Lactate Concentration:** To measure the concentration of lactate in the hippocampal tissue, the Lactate-Glo^TM^ Assay (Promega, Corporation) was used. The experiment was carried out using two independent groups: old animals (O-CTR, n=6), and old animals treated with BAY (O-BAY, n=9). Briefly, the tissue was homogenized and centrifuged as described above, then the supernatant was collected and incubated with the Lactate Detection Reagent for 1 h at room temperature. Then the sample luminescence was measured (Lumi, microdigital). The luminescence intensity was proportional to the concentration of lactate in the samples.
